# Supplementary material for: Microsatellite break-induced replication generates highly mutagenized extrachromosomal circular DNAs
Source: NAR Cancer. 2024 Jun 8;6(2):zcae027. doi: 10.1093/narcan/zcae027 (PMC11161834; doi:10.1093/narcan/zcae027)
Supplement: zcae027_Supplemental_Files [file zcae027_supplemental_files.zip › Supplementary Figure 1 R-upper dTOM.pdf]

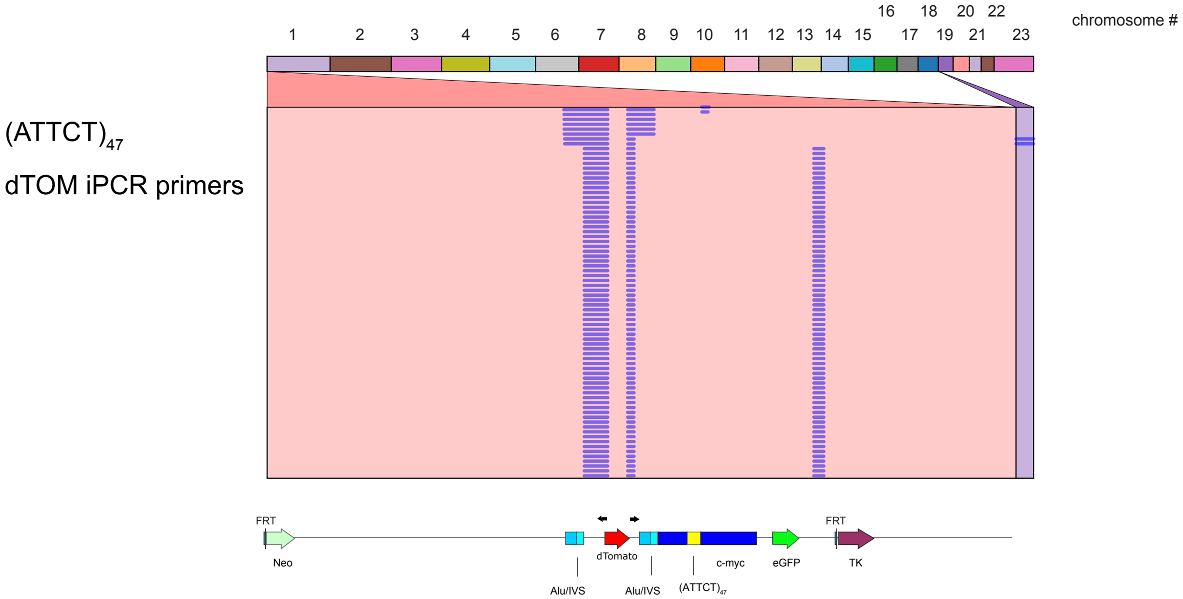

Supplementary Figure 1. Multiple eccDNAs derive from a single ectopic site. iPCR primers flanking the dTOM gene were used to generate 220 reads from $(ATTCT)_{47}$ cells, which are aligned to the ectopic site and chromosome 19. The template switch to chromosome 19 (nt 2,010,376 – 2,010,938) occurred in a 562 bp region with four G4 consensus matches.
