# Supplementary material for: Microsatellite break-induced replication generates highly mutagenized extrachromosomal circular DNAs
Source: NAR Cancer. 2024 Jun 8;6(2):zcae027. doi: 10.1093/narcan/zcae027 (PMC11161834; doi:10.1093/narcan/zcae027)
Supplement: zcae027_Supplemental_Files [file zcae027_supplemental_files.zip › Supplementary Figure 2A-F SnapGene alignments.pdf]

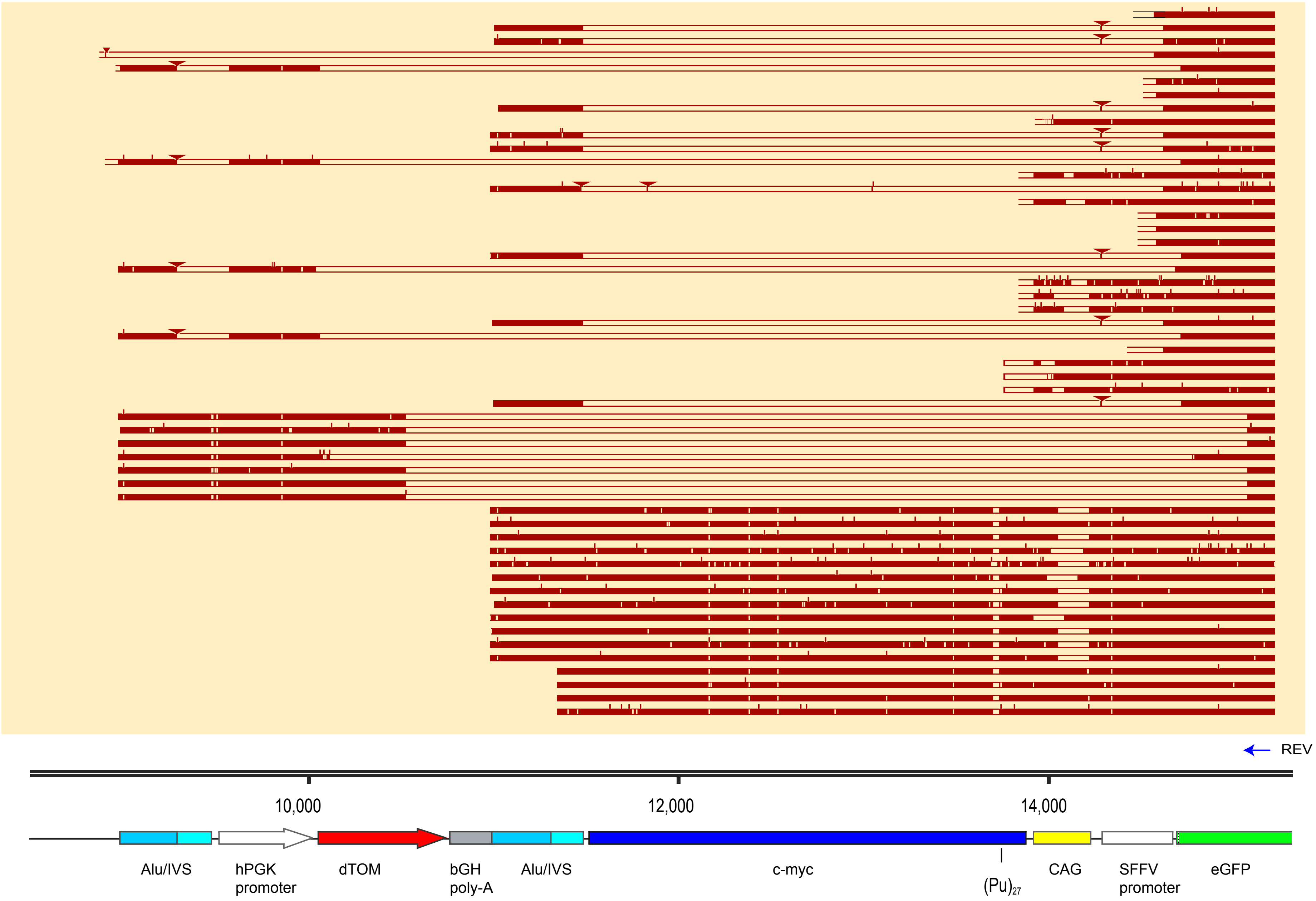

(Supplementary Figure 2 legend follows panel (2F))

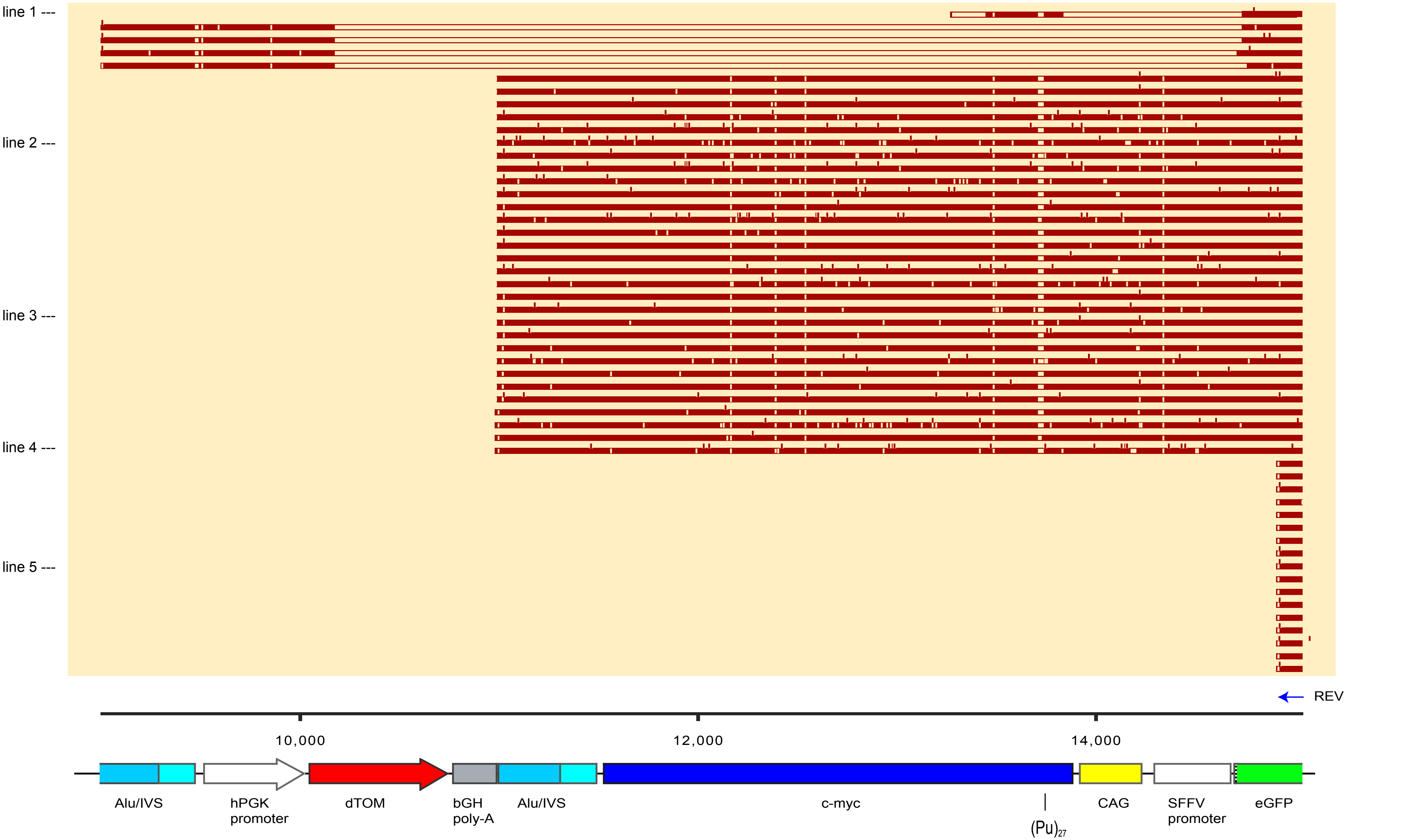

(Supplementary Figure 2 legend follows panel (2F))

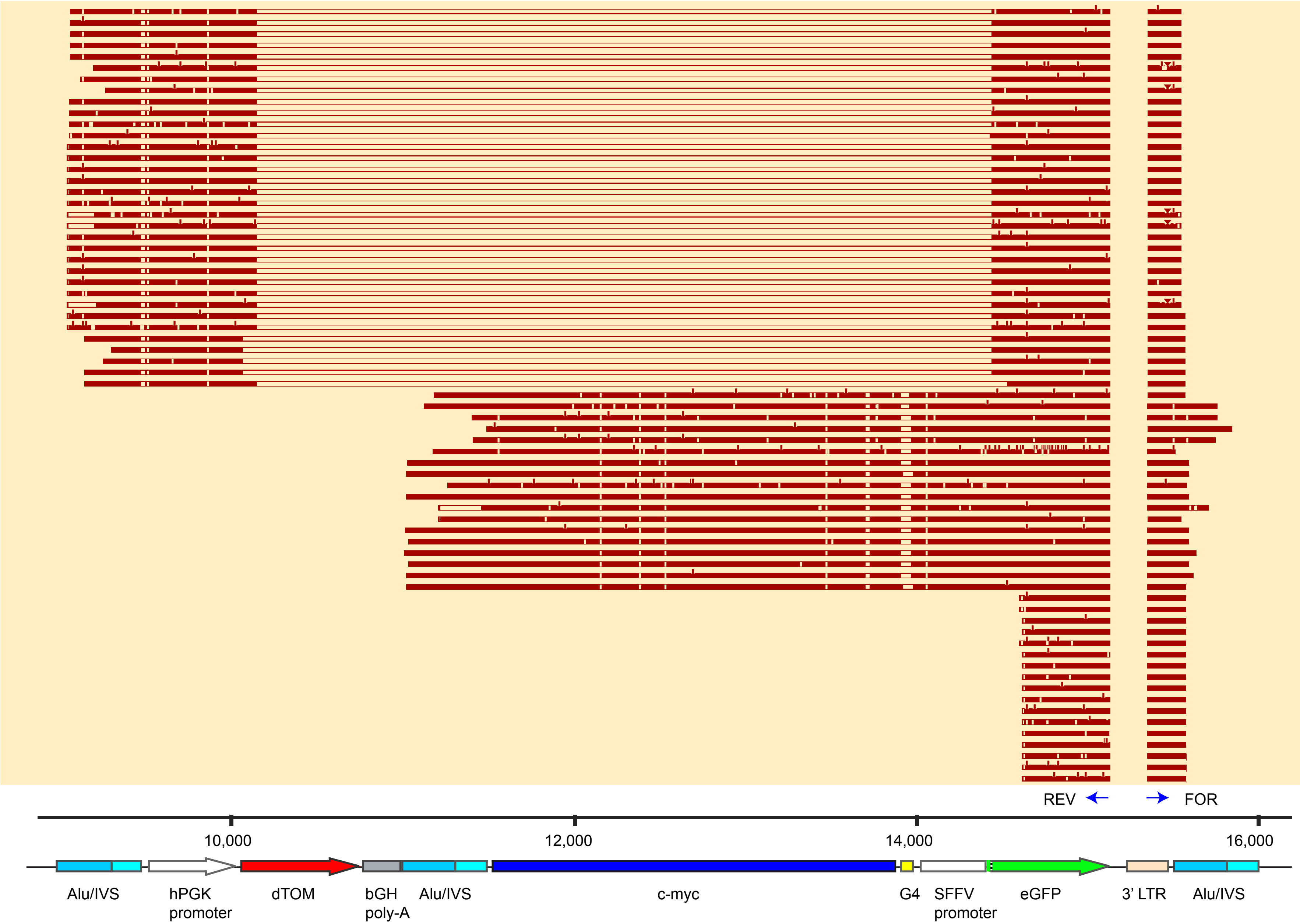

(Supplementary Figure 2 legend follows panel (2F))

Supplementary Figure 2D

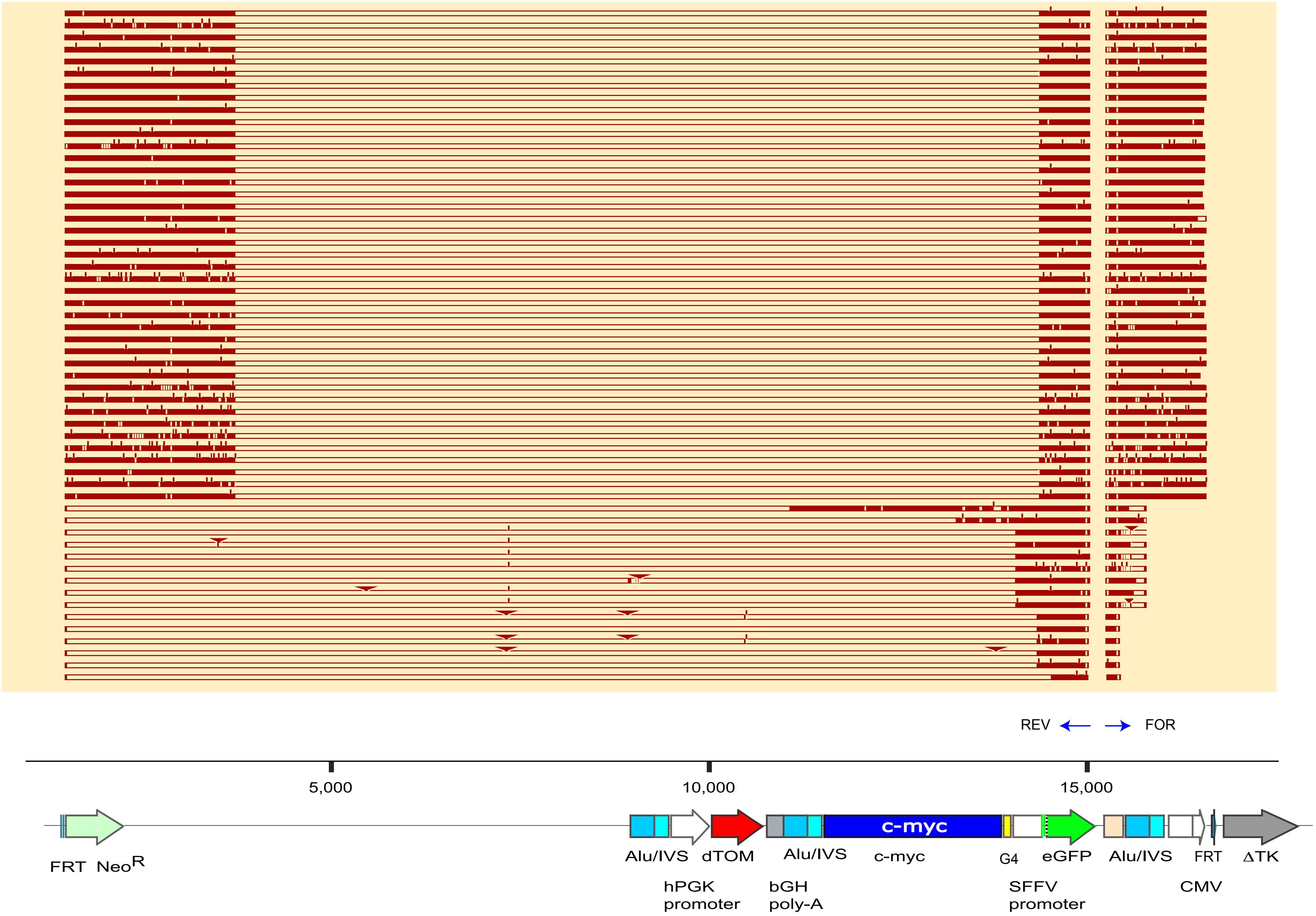

(Supplementary Figure 2 legend follows panel (2F))

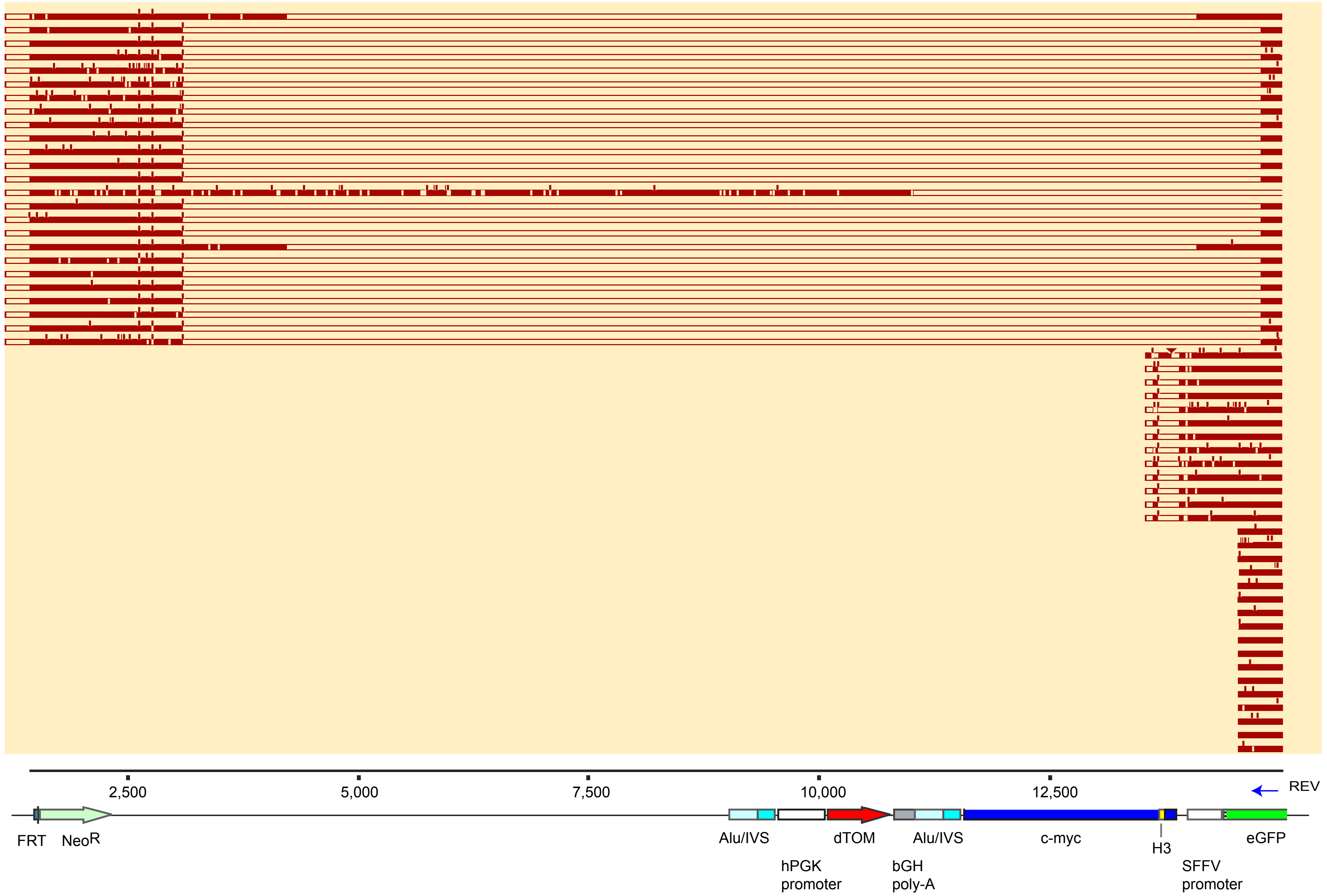

(Supplementary Figure 2 legend follows panel (2F))

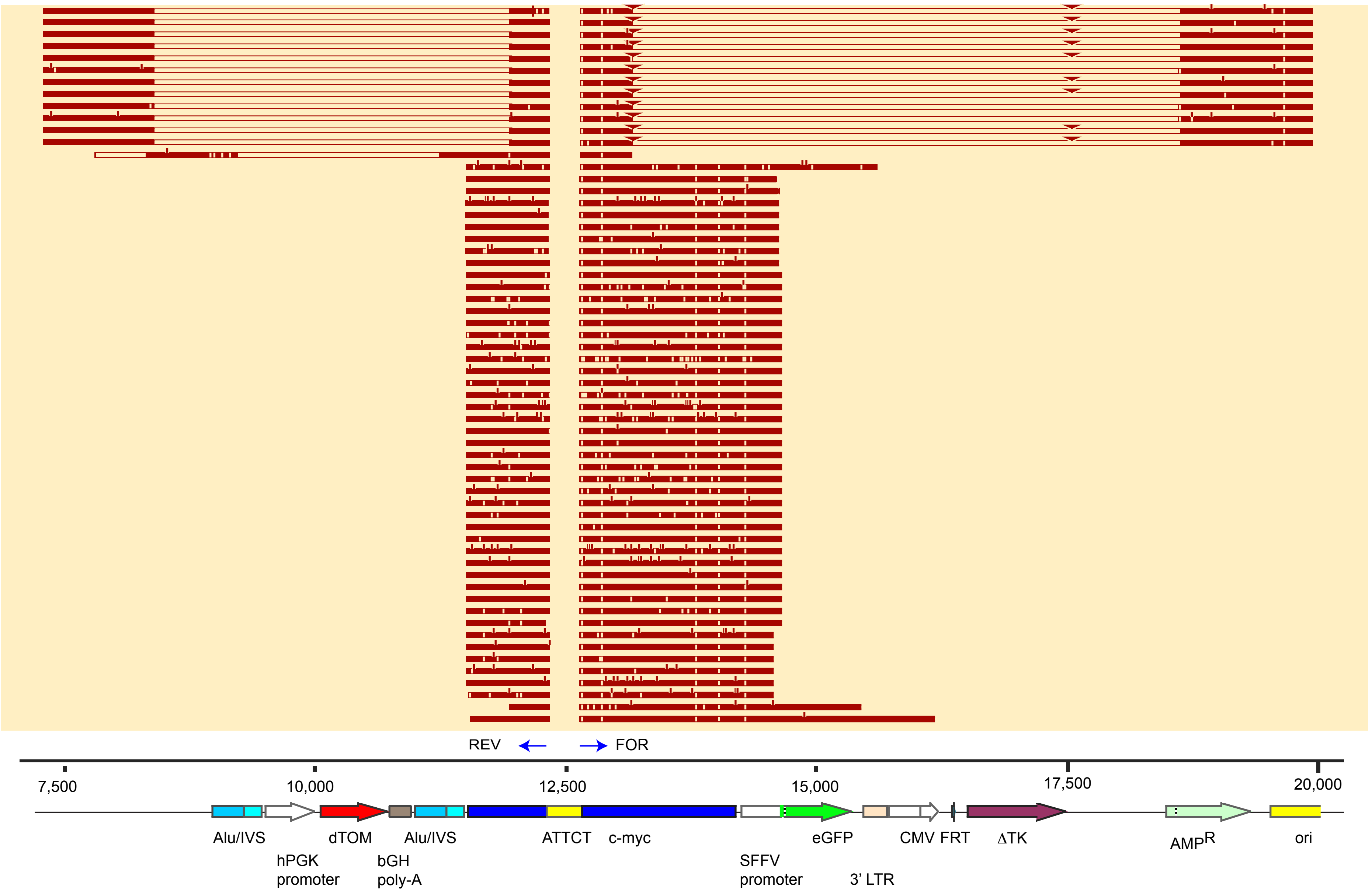

Supplementary Figure 2. eccDNA mutagenesis. Alignment (SnapGene) of sixty reverse iPCR domain reads to the ES map for each non-B DNA cell clone. (A) $(CAG)_{102}$ clone 10 reads (every 440th read from top of Figure 4A) were sampled. (B) $(CAG)_{102}$ clone 13 reads (every 4th read from top of Figure 4B) were sampled. (C) G4 clone 1 reads (every 6th read from top of Figure 4C) were sampled. (D) G4 clone 6 reads (every 28th read from top of Figure 4D) were sampled. (E) H3 clone 10 reads (every 30th read from top of Figure 4E) were sampled. (F) $(ATTCT)_{47}$ clone reads (every 21st read from top of Figure 4F) were sampled. Inverted triangles (not to scale) and upward tics represent insertions; unfilled boxes represent mismatches or deletions (to scale).
