# Supplementary material for: Microsatellite break-induced replication generates highly mutagenized extrachromosomal circular DNAs
Source: NAR Cancer. 2024 Jun 8;6(2):zcae027. doi: 10.1093/narcan/zcae027 (PMC11161834; doi:10.1093/narcan/zcae027)
Supplement: zcae027_Supplemental_Files [file zcae027_supplemental_files.zip › Supplementary Figure 3 CIRCOS.pdf]

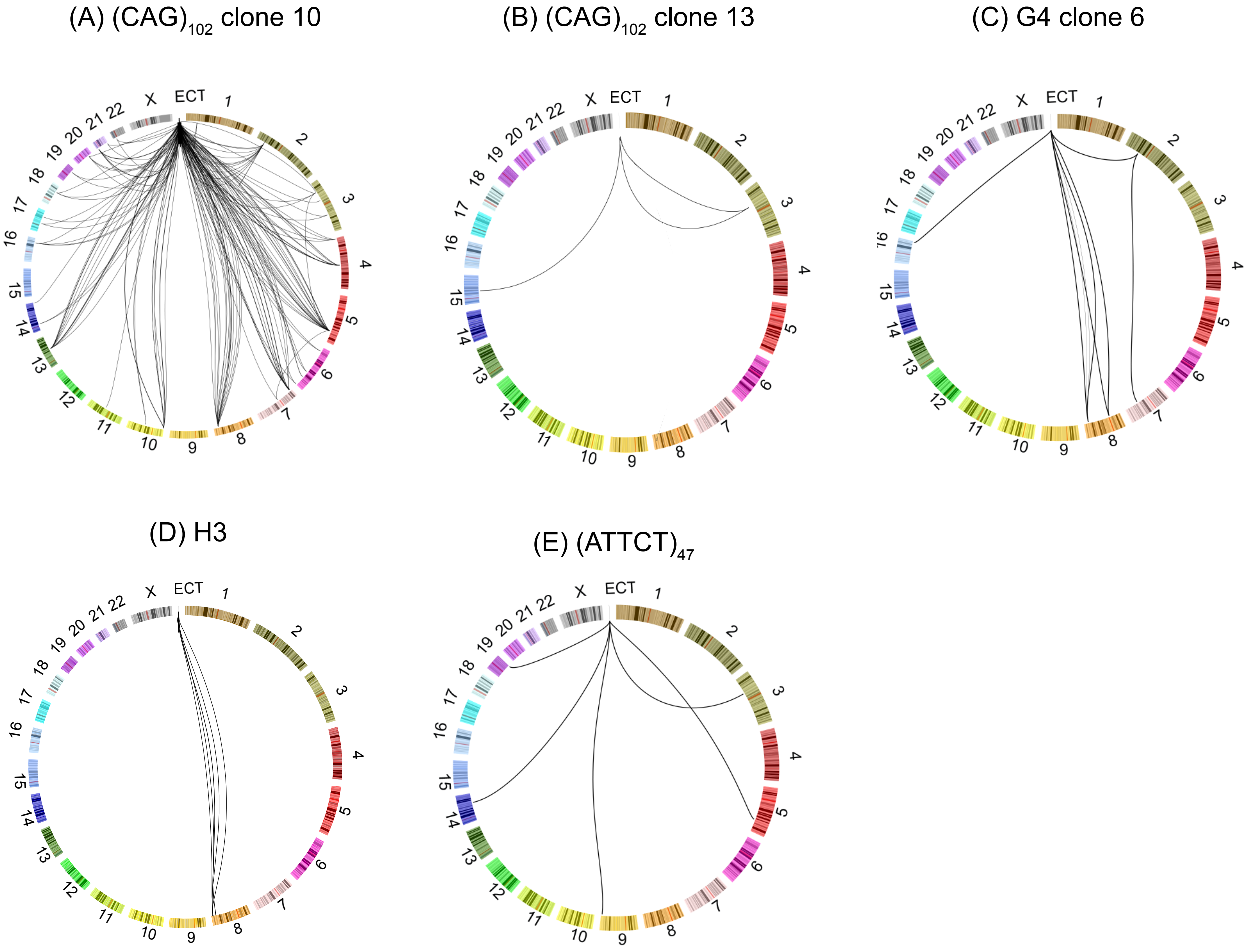

Supplementary Figure 3. Nonallelic template switches. Nonallelic template switches in eccDNAs were visualized using Circos (64). (A) (CAG)$_{102}$ c.10 cell eccDNA, (B) (CAG)$_{102}$ c.13 cell eccDNA, (C) G4, c.6 cell eccDNA, (D) H3 cell eccDNA, (E) (ATTCT)$_{47}$ cell eccDNA; ECT, ES.
