# Supplementary material for: Microsatellite break-induced replication generates highly mutagenized extrachromosomal circular DNAs
Source: NAR Cancer. 2024 Jun 8;6(2):zcae027. doi: 10.1093/narcan/zcae027 (PMC11161834; doi:10.1093/narcan/zcae027)
Supplement: zcae027_Supplemental_Files [file zcae027_supplemental_files.zip › Supplementary Figure 4A-F composite template switching.pdf]

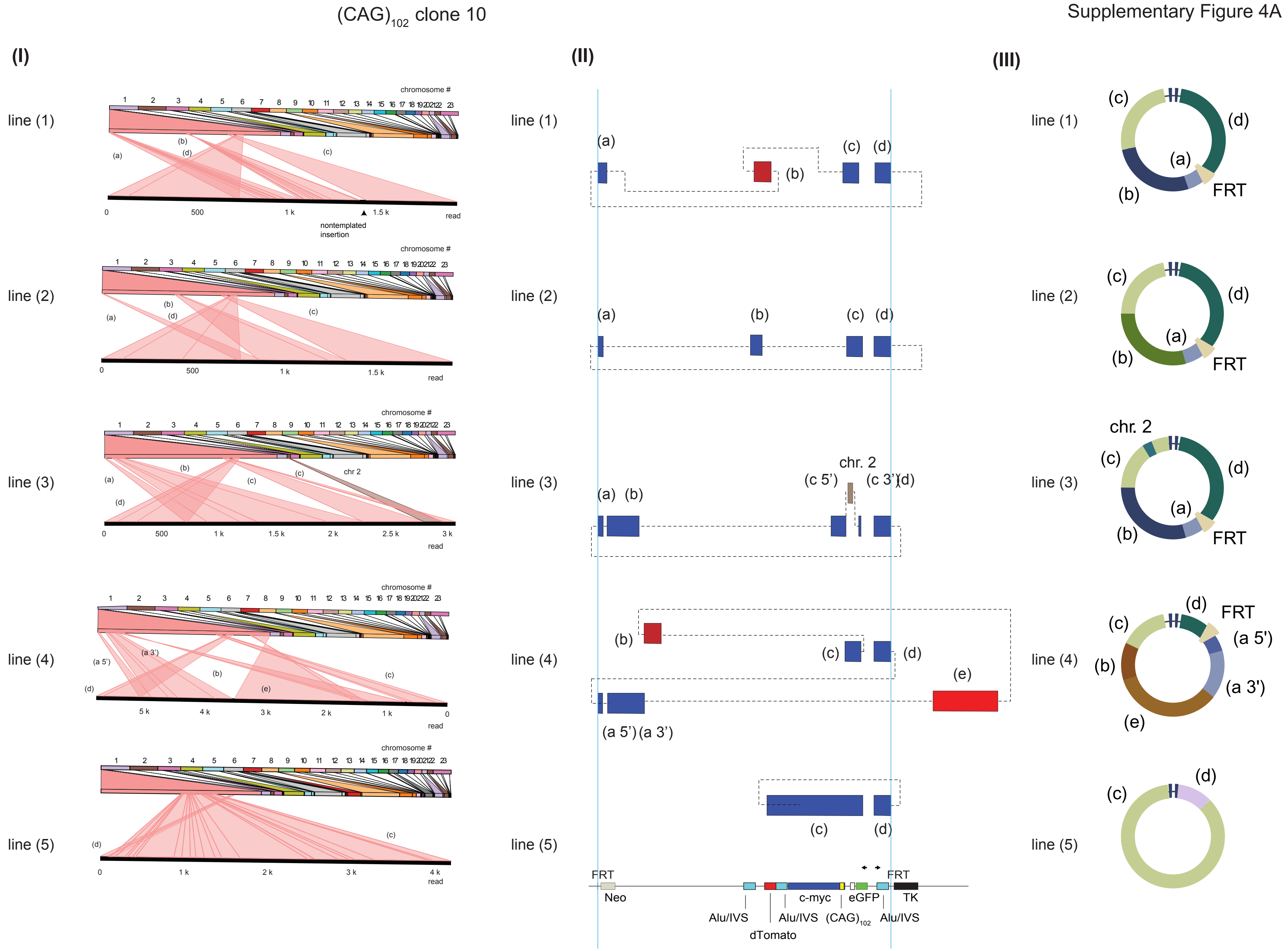

(Supplementary Figure 4 legend follows panel 4F)

$(CAG)_{102}$ clone 13

Supplementary Figure 4B

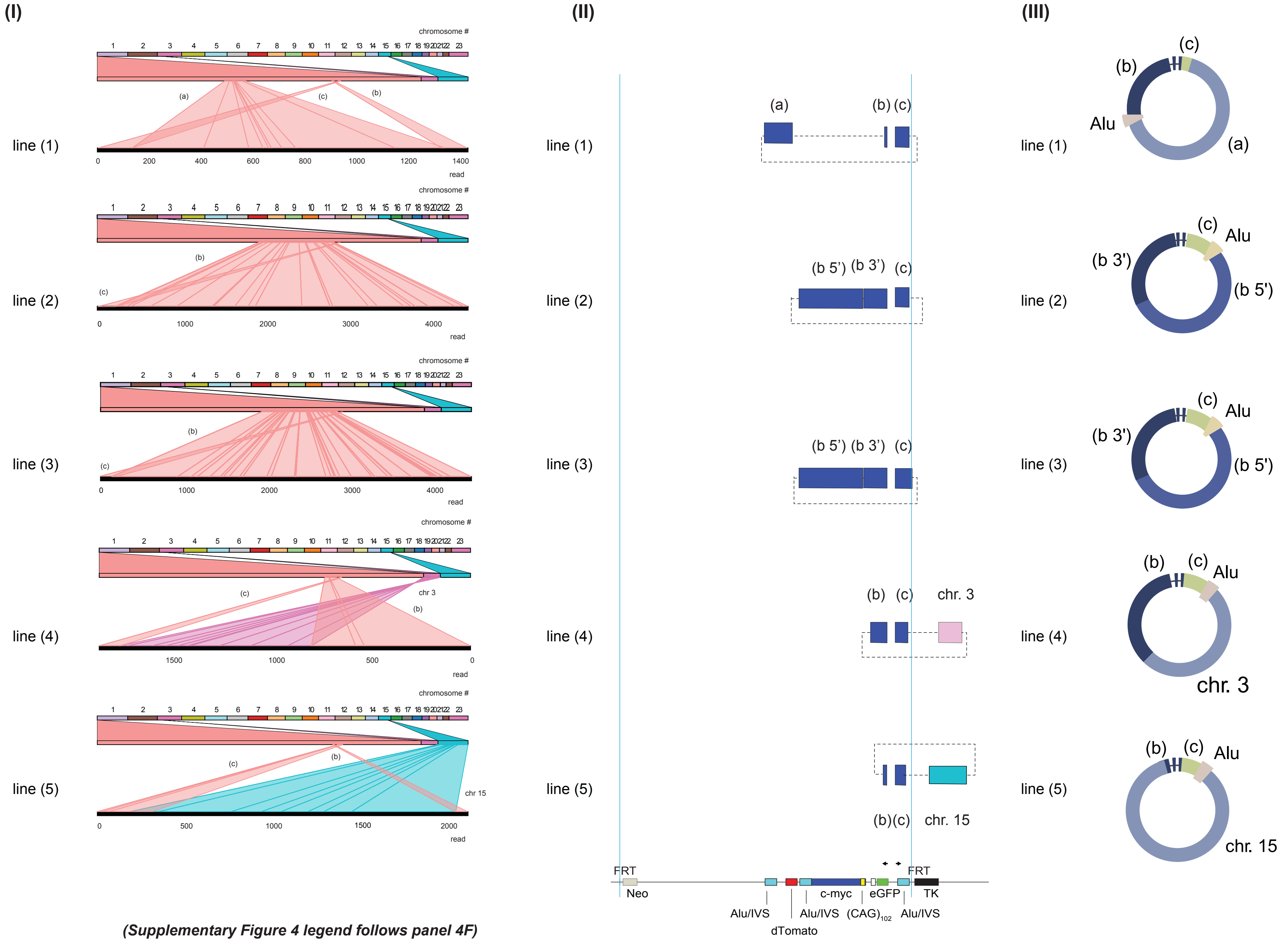

(Supplementary Figure 4 legend follows panel 4F)

Supplementary Figure 4C

G4 clone 1

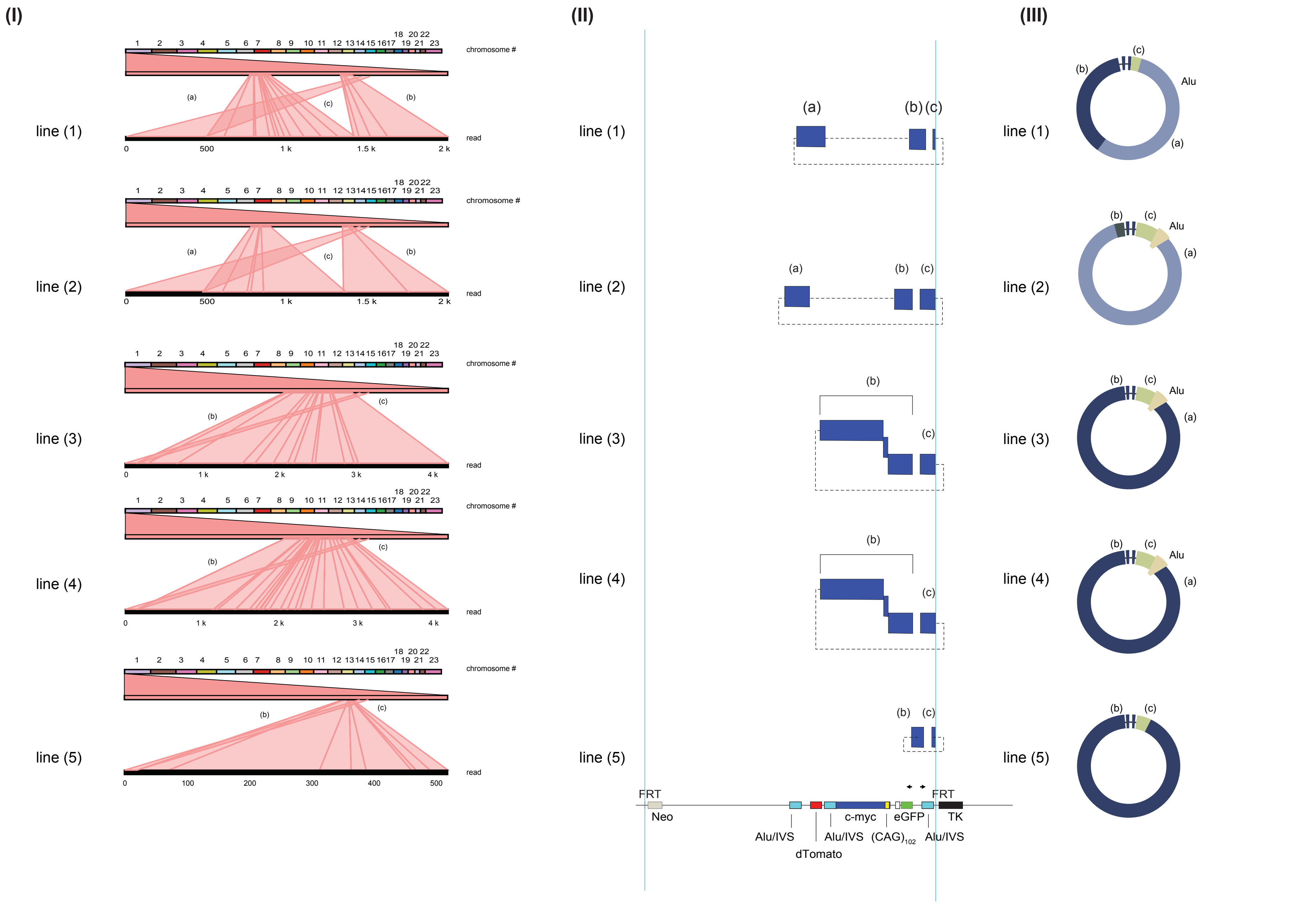

(Supplementary Figure 4 legend follows panel 4F)

Supplementary Figure 4D

G4 clone 6

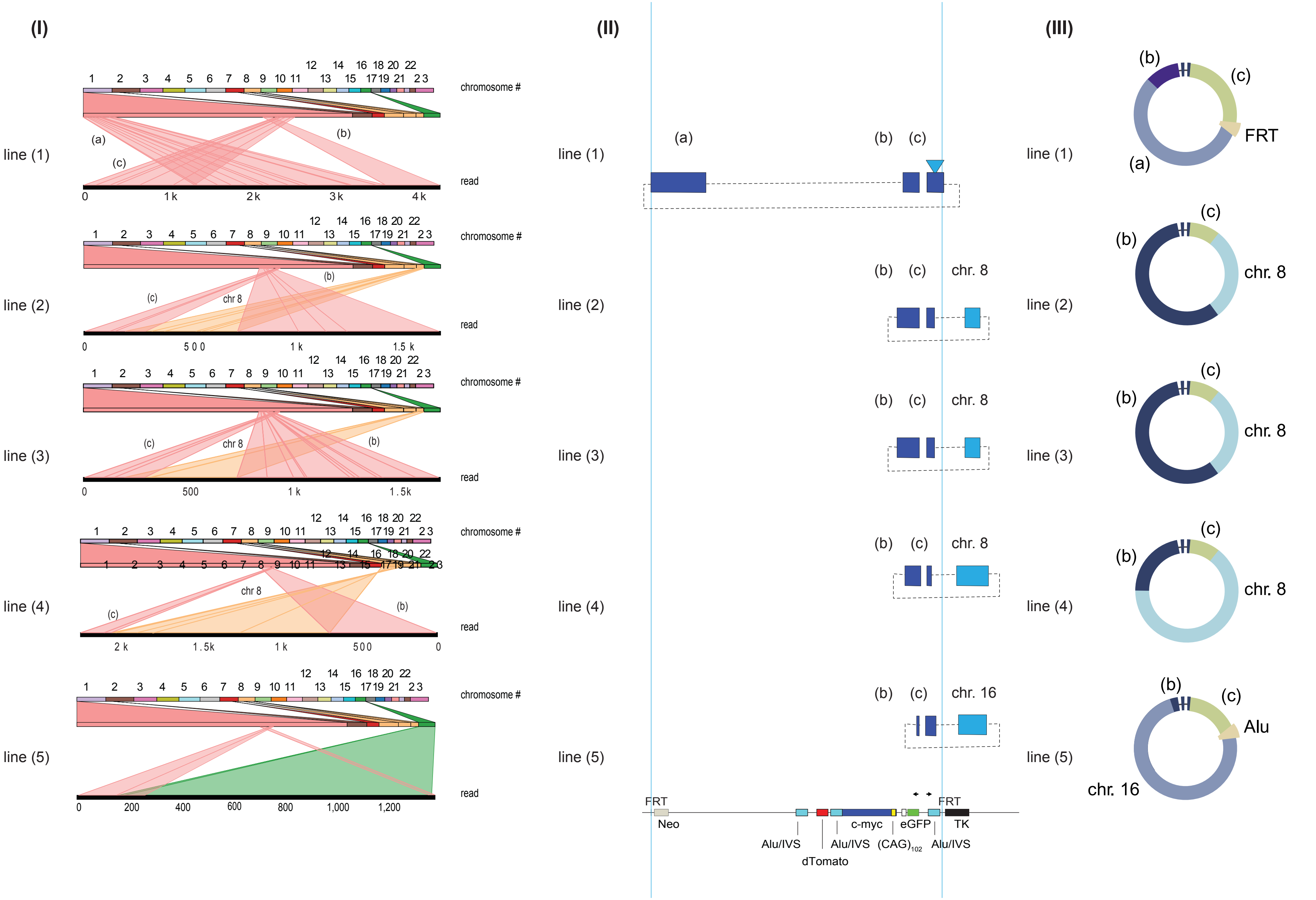

(Supplementary Figure 4 legend follows panel 4F)

Supplementary Figure 4E

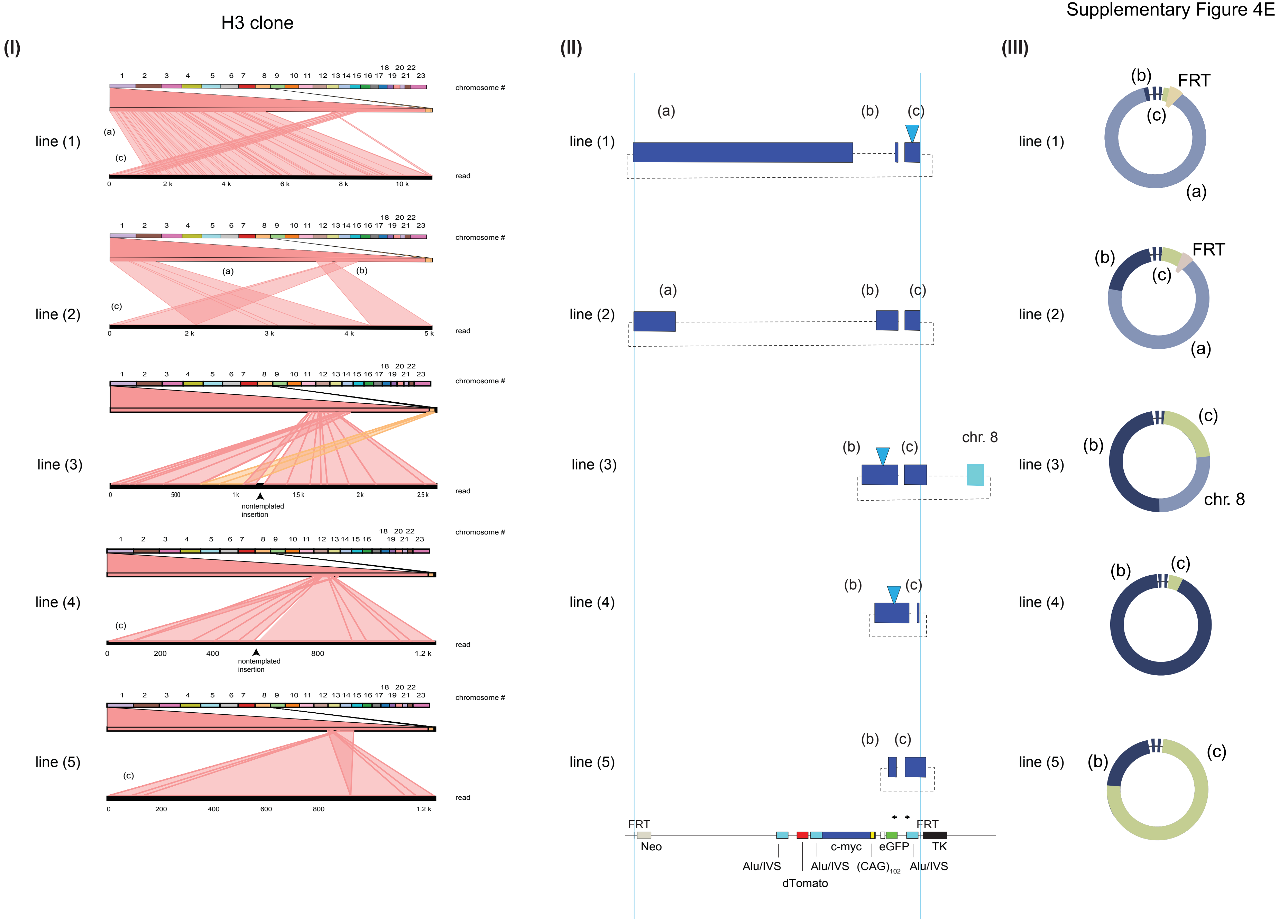

(Supplementary Figure 4 legend follows panel 4F)

Supplementary Figure 4F

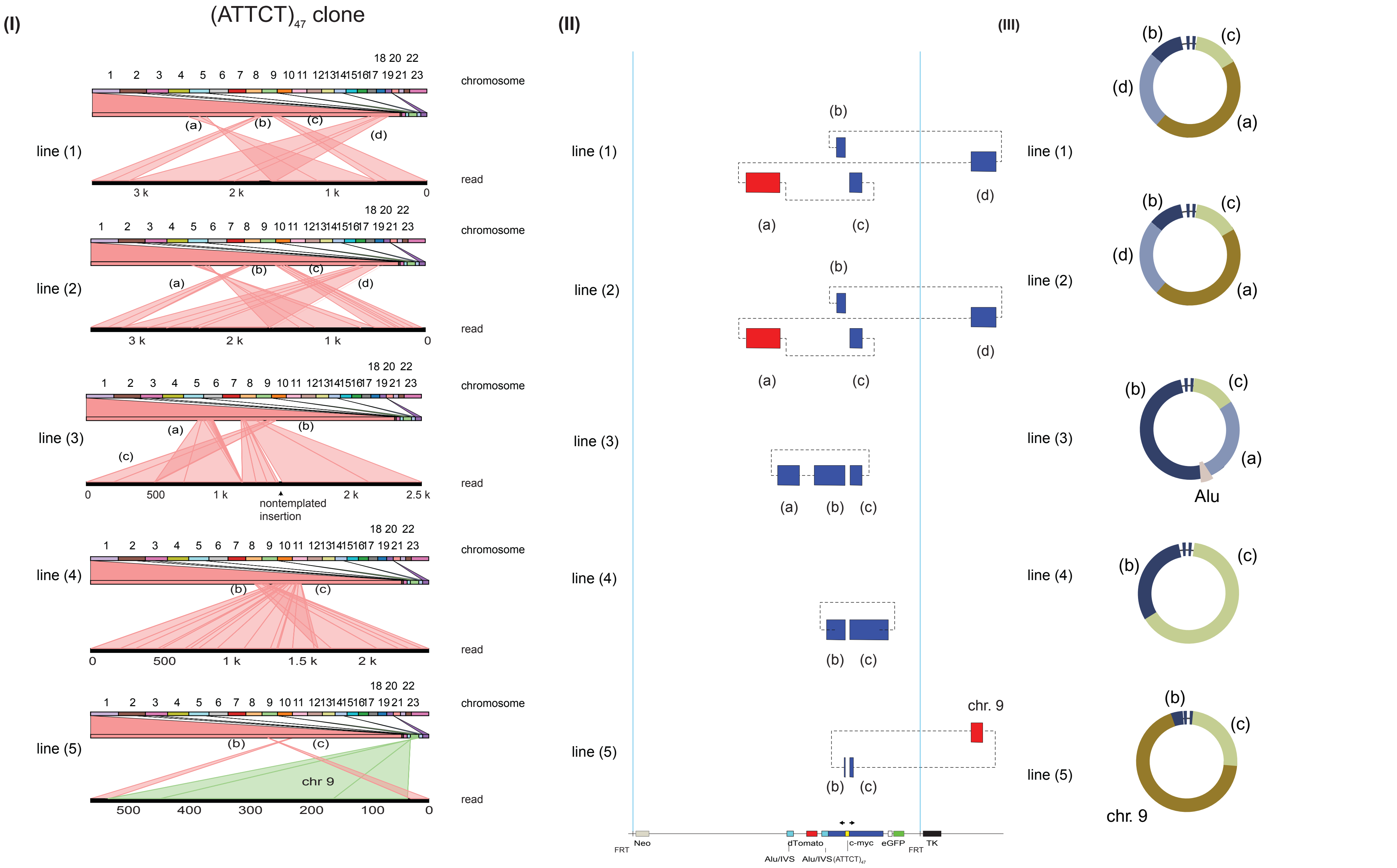

Supplementary Figure 4. Template switching patterns of eccDNAs. Individual read lines (Figure 4) were mapped (A) $(CAG)_{102}$ clone 10, (B) $(CAG)_{102}$ clone 13, (C) G4 clone 1, (D) G4 clone 6, (E) H3, (F) $(ATTCT)_{47}$. **(I)** In the *Query Viewport* view of Ribbon. Chromosome numbers are shown at the top; the lower heavy black line in each panel is the complete read. Heavier red lines within a domain indicate indels. Letters (a) - (e) and chromosome designations correspond to template switching domains of Figure 4. **(II)** Diagrammed to show template switching of ES domains. **(III)** Diagrammed to show the relative structures of the eccDNA circles.
