# Supplementary material for: Microsatellite break-induced replication generates highly mutagenized extrachromosomal circular DNAs
Source: NAR Cancer. 2024 Jun 8;6(2):zcae027. doi: 10.1093/narcan/zcae027 (PMC11161834; doi:10.1093/narcan/zcae027)
Supplement: zcae027_Supplemental_Files [file zcae027_supplemental_files.zip › Supplementary Figure 5A-D high mutation threshold.pdf]

Supplementary Figure 5A, B

(A) $(CAG)_{102}$ clone 10
no mutation threshold

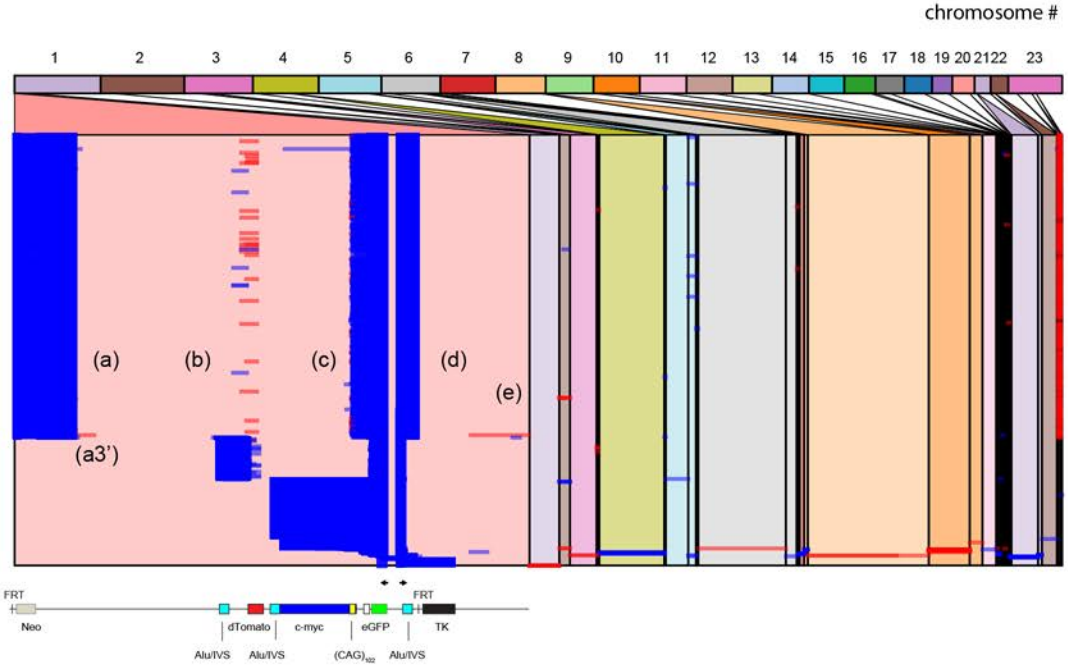

(B) $(CAG)_{102}$ clone 10
high mutation threshold

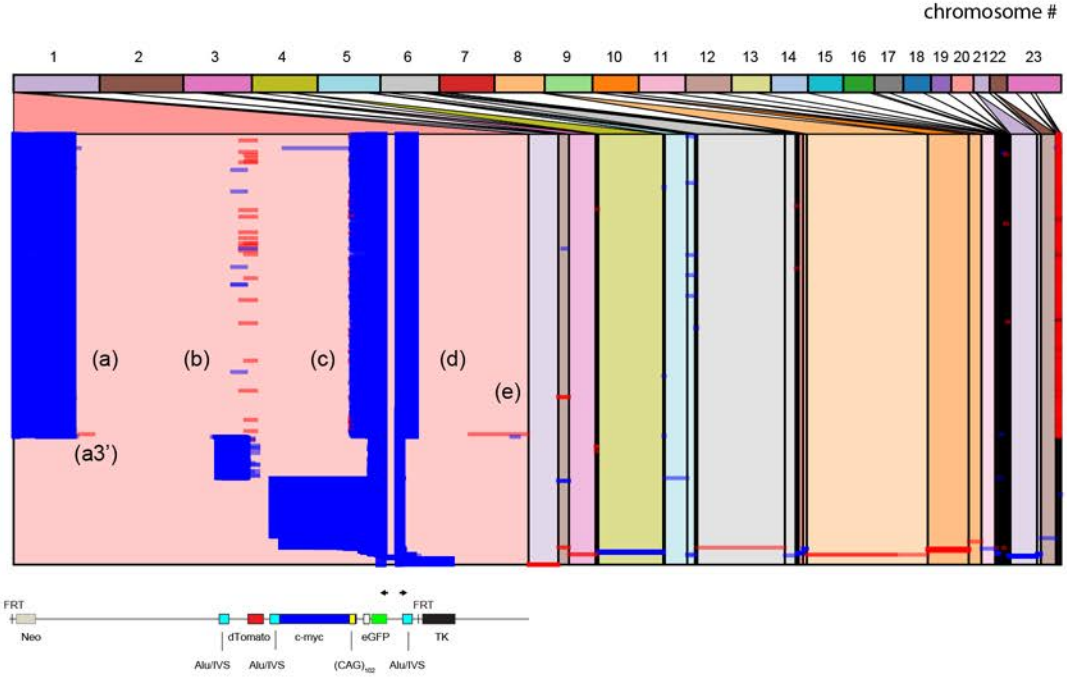

(C) $(CAG)_{102}$ clone 13
no mutation threshold

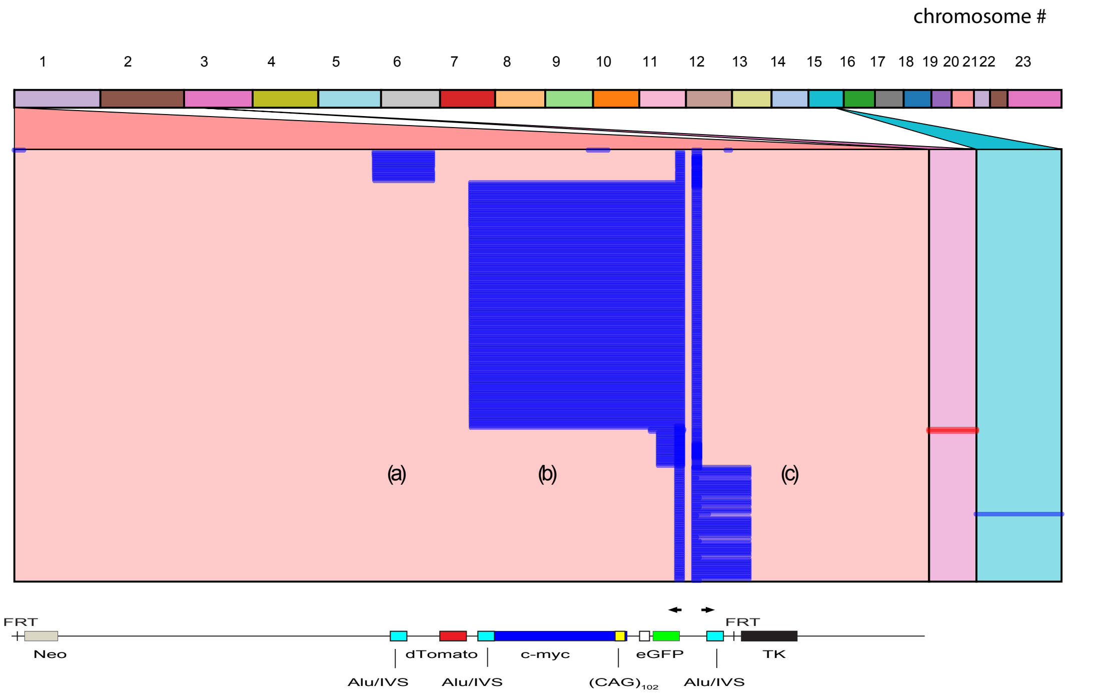

(D) $(CAG)_{102}$ clone 13
hgh mutation threshold

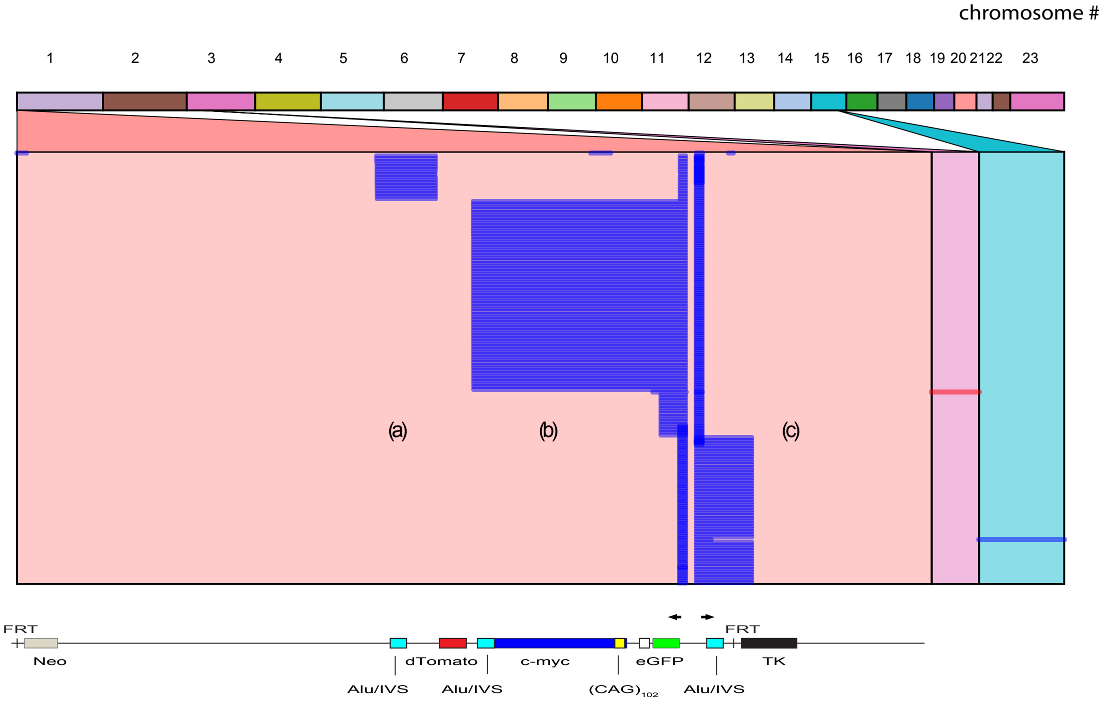

Supplementary Figure 5. Application of a high mutation threshold to $(CAG)_{102}$ eccDNA alignments. Alignment of $(CAG)_{102}$ clone 10 reads without (A) or with (B) a mutation of threshold of 2.0 mutations (indels, base substitutions) per kb. Alignment of $(CAG)_{102}$ clone 13 reads without (C) or with (D) a mutation of threshold of 2.0 mutations (indels, base substitutions) per kb.
