# Supplementary material for: Microsatellite break-induced replication generates highly mutagenized extrachromosomal circular DNAs
Source: NAR Cancer. 2024 Jun 8;6(2):zcae027. doi: 10.1093/narcan/zcae027 (PMC11161834; doi:10.1093/narcan/zcae027)
Supplement: zcae027_Supplemental_Files [file zcae027_supplemental_files.zip › Supplementary Figure 6A-F ALVIS ATTCT.pdf]

Supplementary Figure 6A

$(CAG)_{102}$ clone 10

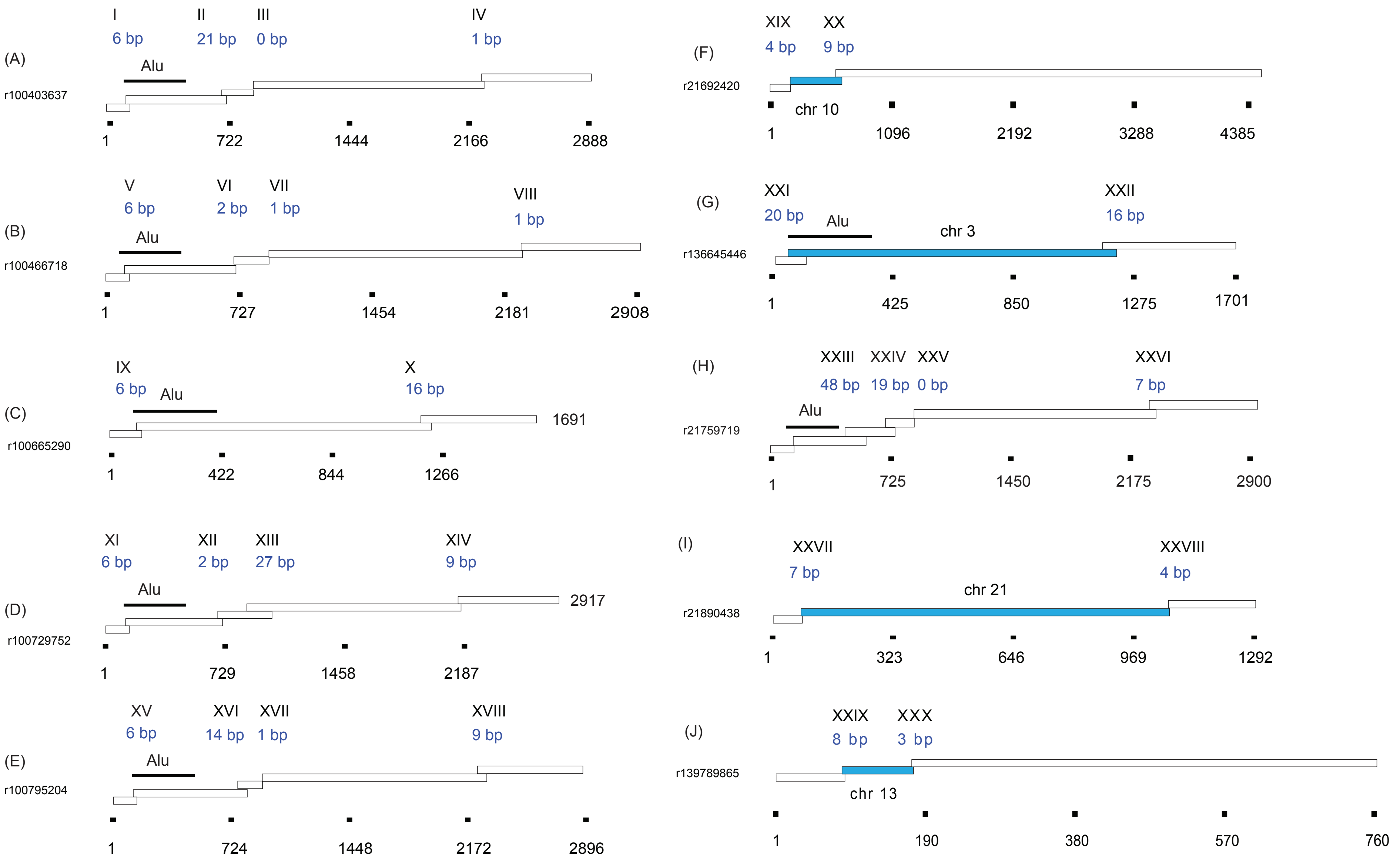

(Supplementary Figure 6 legend follows panel 6F)

Supplementary Figure 6B

(CAG)$_{102}$ clone 13

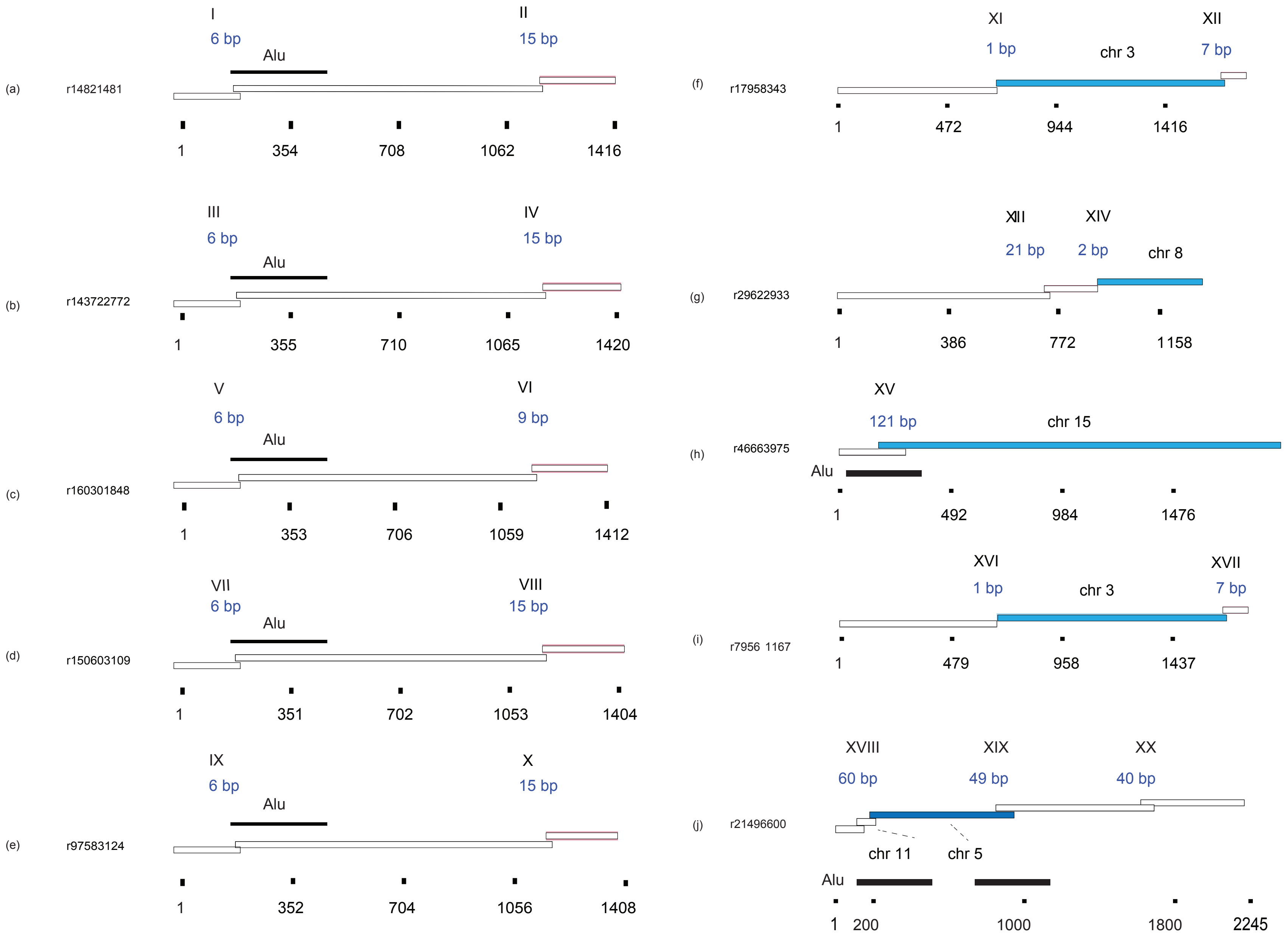

(Supplementary Figure 6 legend follows panel 6F)

Supplementary Figure 6C

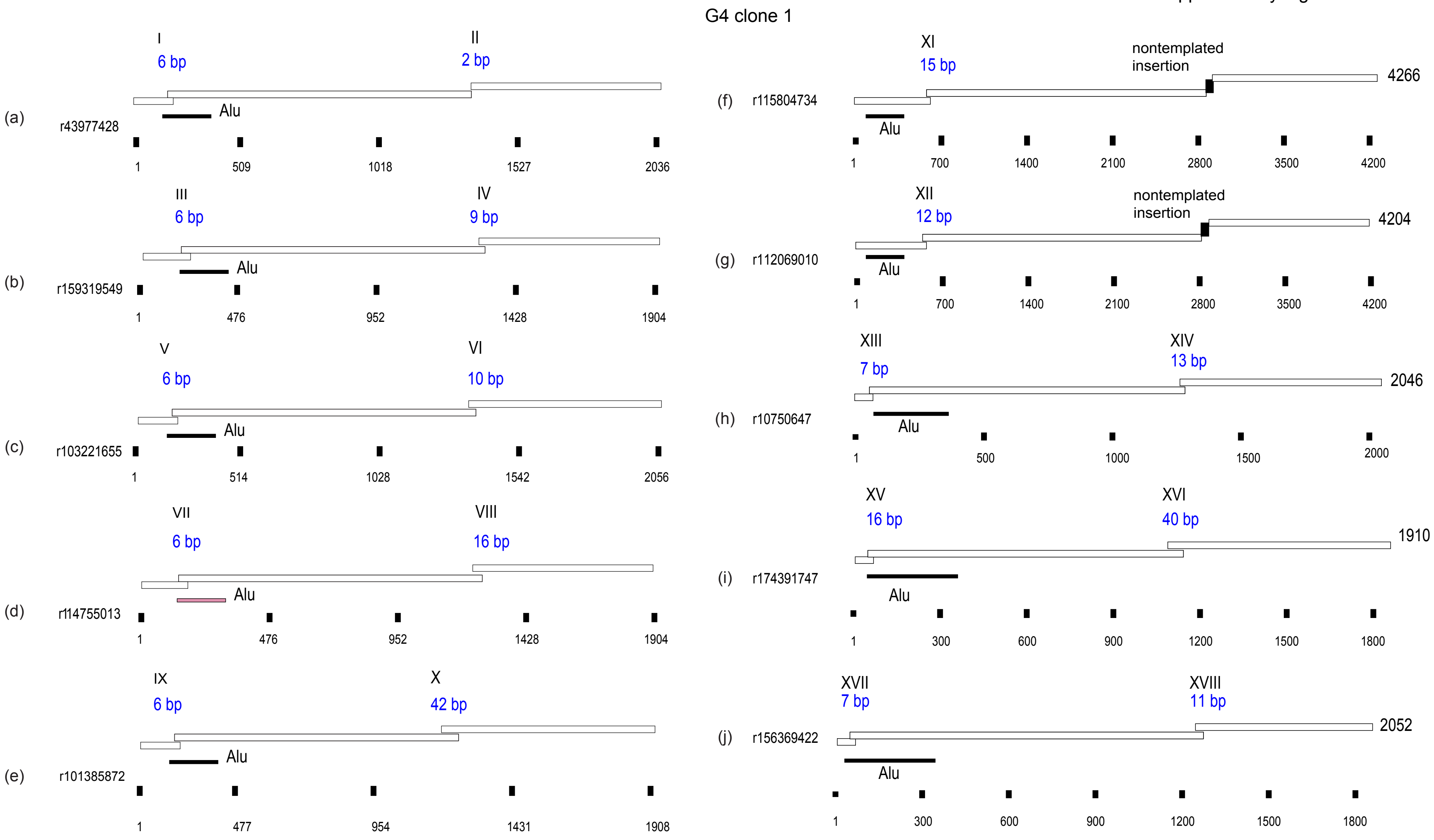

(Supplementary Figure 6 legend follows panel 6F)

G4 clone 6

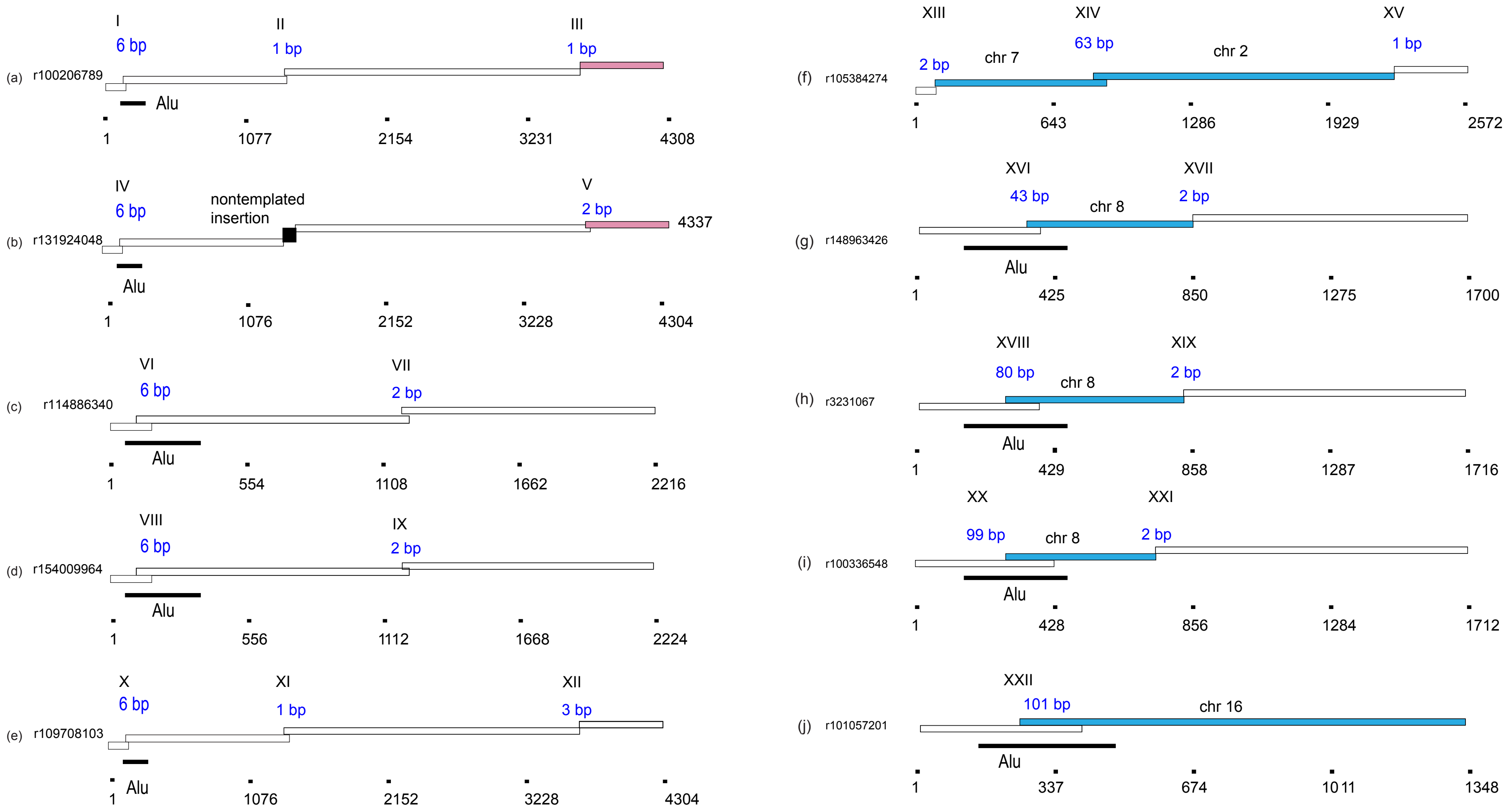

(Supplementary Figure 6 legend follows panel 6F)

Supplementary Figure 6E

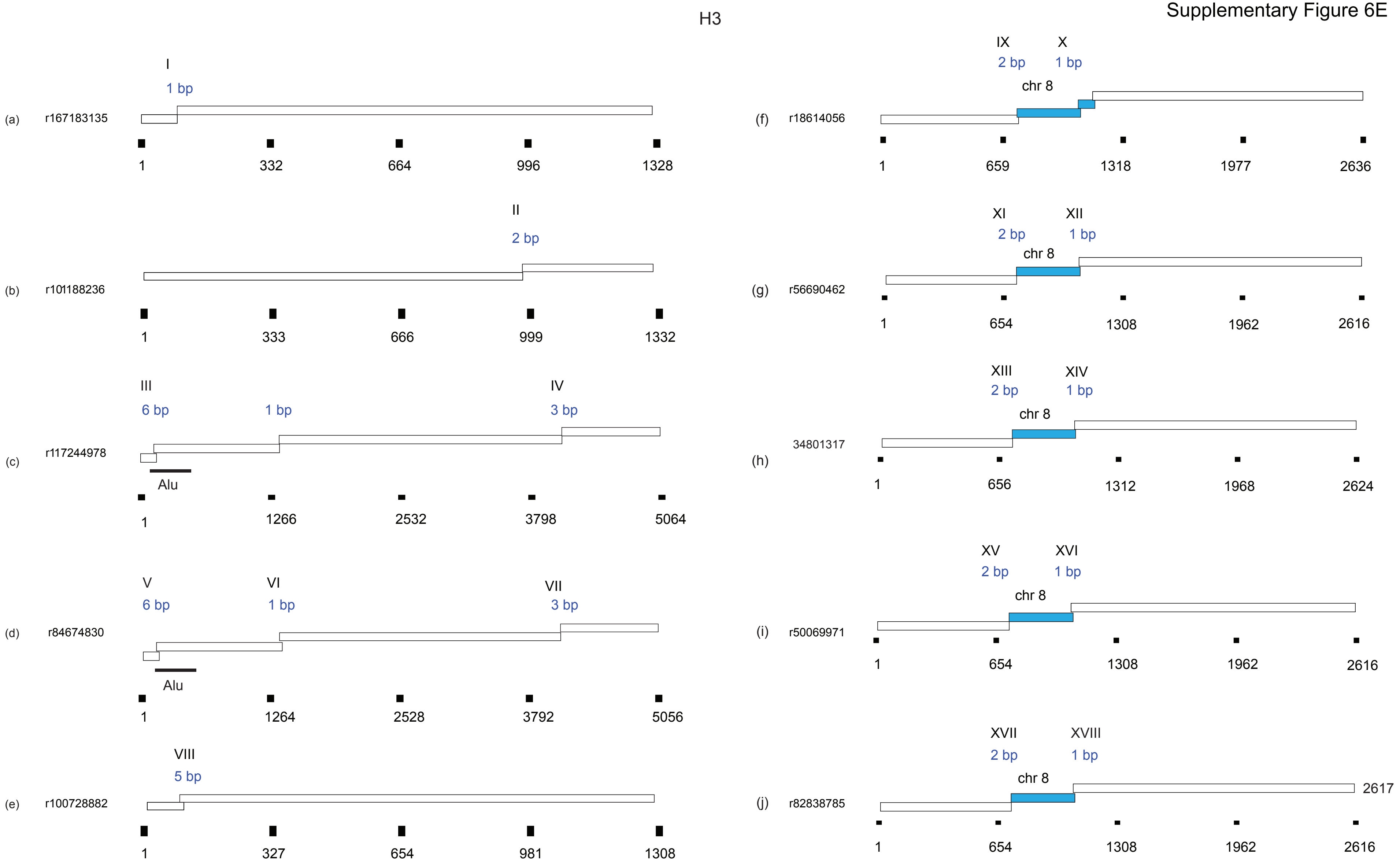

(Supplementary Figure 6 legend follows panel 6F)

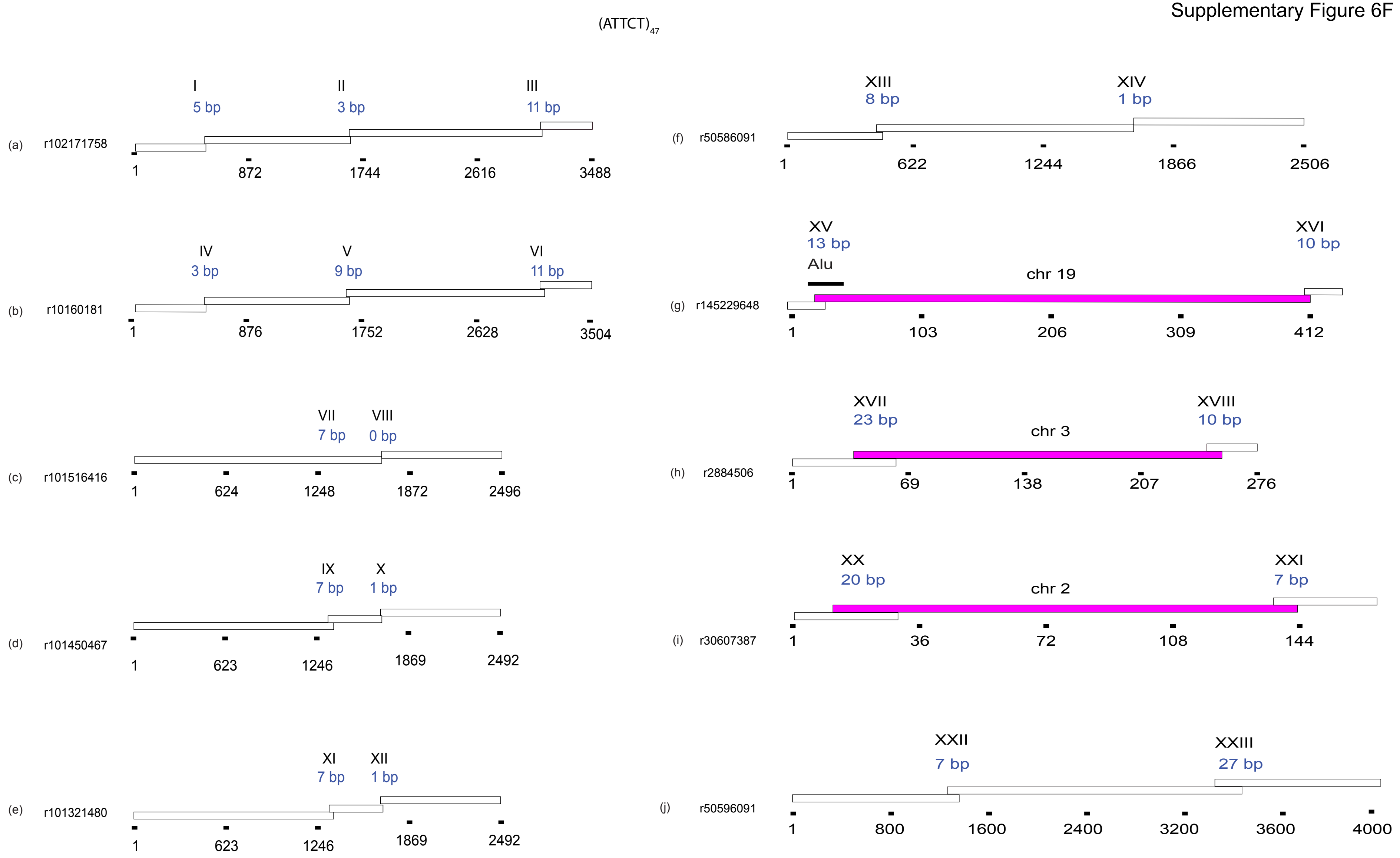

Supplementary Figure 6. Microhomology overlaps. (a)-(j) are eccDNA reads, distinct from lines 1-5, representative of clades from (A) $(CAG)_{102}$ clone 10 cells, (B) $(CAG)_{102}$ clone 13 cells, (C) G4 clone 1 cells. (D) G4 clone 6 cells, (E) H3 cells, (F) $(ATTCT)_{47}$ cells. Each box is a template switch domain. Blue domains are nonallelic template switches. Roman numerals and blue base pair numerals are sequence overlaps. Approximate length coordinates are shown below (or alongside) each read. Analysis was performed with ALVIS (192) and reformatted for presentation.
