# Supplementary material for: Microsatellite break-induced replication generates highly mutagenized extrachromosomal circular DNAs
Source: NAR Cancer. 2024 Jun 8;6(2):zcae027. doi: 10.1093/narcan/zcae027 (PMC11161834; doi:10.1093/narcan/zcae027)
Supplement: zcae027_Supplemental_Files [file zcae027_supplemental_files.zip › Supplementary Figure 7A-F Template overlaps.pdf]

Supplementary Figure 7A

CAG clone 10 Line 1

(FRT/FRT junction)

Domain d 2 --- GAAATTC GAAGTTCCTA TTCCGAAGTT CCTATTCTCT AGAAAGTATA GGAACTTC 724
Domain a 677 [ES 16,342] GAAGTTCCTA TTCCGAAGTT CCTATTCTCT AGAAAGTATA GGAACTTCACCAGAT --- 1,063

Domain a 677 --- ACCTTGCTCCTGCC 1063 [ES 1,833]
Domain b 1,062 [ES 1,832] CCTCGGG --- 1,649

Domain b 1,061 --- CGGCCGCTGCT CTCGGCGCGC C 1,649 [ES 9,887]
Domain c 1,639 [16,350] CTCGGCGCGC CAGTCCTCCGA --- 2,238

CAG clone 10 line 2

(FRT/FRT junction)

Domain d 2 --- ATTC GAAGTTCCTA TTCCGAAGTT CCTATTCTCT AGAAAGTATA GGAACTTC 728 [ES 16,398]
Domain a 681 [ES 1,446] GAAGTTCCTA TTCCGAAGTT CCTATTCTCT AGAAAGTATA GGAACTTCAC CAATTGAA --- 892

Domain a 681 --- CAGACAATCGGCTGCTCTG 834 [ES 1599]
Domain b 824 [ES 9572] CGGCTGCTCTGGGCGTGGTTCCGG --- 1,327

Domain b 824 ---CCAGGGGG ATCCACCGGT CGCCACCATG GTGAGCAAGG GCGAGGAG 1,327 [ES 10,073]
Domain c 1,287 [ES 14,677] GGG ATCCACCGGT CGCCACCATG GTGAGCAAGG GCGAGGAG CTGTTC --- 1,835

CAG clone 10 line 3

(FRT/FRT junction)

Domain d 2 --- AAATTC GAAGTTCCTA TTCCGAAGTT CCTATTCTCT AGAAAGTATA GGAACTTC 727 [ES 16,397]
Domain a 680 [ES 1,446] GAAGTTCCTA TTCCGAAGTT CCTATTCTCT AGAAAGTATA GGAACTTCAC CAGATC --- 898

Domain a 680 --- AAGACCGACCTGTCC 898 [ES 1,664]
Domain b 898 [ES 1,664] CACGAGATTTCGATTCCA --- 2,272

Domain b 898 --- ATTACGCGCGCT 2272[ES 3,718]
Domain c 5’ 2,265 [ES 14,578] CGCGCGCTTCTGCTTCCCGAGCTCTATAAAAGAGC --- 2,800

Domain c 5’ 2,265 --- GACTTCAAGGAGGACGGCAACATCCTGGGG 2,800 [ES 15,113]
Chr. 2 [nt 32916352] GGGGGGAGGG GGGGAGGGGG $(G)_{135}$ GGGGGGGG

Chr. 2 [nt 32916352] --- GGGGGGAGGG GGGGAGGGGG $(G)_{135}$ GGGGGGGGG [nt 32916551]
Domain c 3’ 2,956 [ES 15,110] GGGGCACAAGCTG --- 3,072

CAG clone 10 line 4

Domain c 1 --- CGGTGGAGCT 697 [ES 14,560]
Domain b 695 [ES 3,683] GCTCCAATTC --- 1,433

Domain b 695 --- GGAGCTTGTA TCCGCTCATG AGA 1,433 [ES 4,418]
Domain e 1,349 [ES 18,365] TGTA TCCGCTCATG AGACAATAAC --- 3,551

Domain e 1,349 --- CAAAAGCTGG 3,551 [ES 20,568]
Domain a 3’ 3,549 [ES 3,685] TGGGTACCGG --- 4,890

Domain a 3’ 3,549 --- AAATCTCGTG 4,890 [ES 2,349]
Domain a 5’ 4,890 [ES 1,664] GGACAGGTCG --- 5108

(FRT/FRT junction)

Domain a 5’ 4,890 --- GAAGTTCCTA TACTTTCTAG AGAATAGGAA CTTCGGAATA GGAACTTC 5,108 [ES 1,446]
Domain d 5,061 [ES 16,397] GAAGTTCCTA TACTTTCTAG AGAATAGGAA CTTCGGAATA GGAACTTC GAATT --- 5,786

CAG clone 10 line 5

(Alu/Alu junction)

Domain d 1 --- GTGTT TTTTTTTTTT TTTTTTTTTT TTTTTTTTGA GACGGAGTCT CGCTCTGTCG CCCAGGCTGG
Domain a 112 [ES 15,778] TTTTTTTTTT TTTTTTTTTT TTTTTTTTGA GACGGAGTCT CGCTCTGTCG CCCAGGCTGG

AGTGCAGTGG CGGGATCTCG GCTCACTGCA AGCTCCGCCT CCCGGGTTCA CGCCATTCTC CTGCCTCAGC CTCCCAAGTA
AGTGCAGTGG CGGGATCTCG GCTCACTGCA AGCTCCGCCT CCCGGGTTCA CGCCATTCTC CTGCCTCAGC CTCCCAAGTA

GCTGGGACTA CAGGCGCCCG CCACTACGCC CGGC 284 [ES 15,942]
GCTGGGACTA CAGGCGCCCG CCACTACGCC CGGCCTAAAT --- 4,001

<u>CAG clone 13 line 1</u>

```
(Alu/Alu junction)            *                                                *
Domain b       1 --- CACG TTTTTTTTTT TTTTTTTTTT TTTTTTTTTG AGACGGAGTC TCGCTCTGTC GCCCAGGCTG
Domain a   135 {ES 8,984] TTTTTTTTTT TTTTTTTTTT TTTTTTTTTG AGACGGAGTC TCGCTCTGTC GCGGGATCTC
                                       *                     *
GAGTGCAGTG GCCCAGGCTG GGCTCACTGC AAGCTCCGCC TCCCGGGTTC ACGCCATTCT CCTGCCTCAG CCTCCCAAGT
GAGTGCAGTG GCGGGATCTC GGCTCACTGC AAGCTCCGCC TCCCGGGTTC ACGCCATTCT CCTGCCTCAG CCTCCCAAGT

                                                               **
AGCTGGGACT ACAGGCGCCC GCCACTACGC CCGGCTAATT TTTTGTATTT TTAGTAGAGA CGGGGTTTCA CCGTTTTAGC
AGCTGGGACT ACAGGCGCCC GCCACTACGC CCGGCTAATT TTTTGTATTT TTAGTAGAGA CGGGGTTTCA CCGTTTTAGC

CGGGATGGTC TCGATCTCCT GACCTCGTGA TCCGCCCGCC TCGGCCTCCC AAAGTGCTGG GATTACAGGC GTGAGCCACC
CGGGATGGTC TCGATCTCCT GACCTCGTGA TCCGCCCGCC TCGGCCTCCC AAAGTGCTGG GATTACAGGC GTGAGCCACC
                      *                 *                    *
GCGCCCGGCC TAAATAATGT TTTTTAGATT TGCCAAATTT GTACTGAACT CTAGAATGAC TAGTTAATCT TTTTCTCCTA
GCGCCCGGCC TAAATAATGT TTTTTAGATT TGCCAAATTT GTACTGAACT CTAGAATGAC TAGTTAATCT TTTTCTCCTA
                  *                                *
TTGCAACCTT CTAAATAATC ATTCAGCAAA ATAGCCTAAT AGCTCAACTA ACAGTAGATG TTTCATAACA G 451 [ES 16,232]
TTGCAACCTT CTAAATAATC ATTCAGCAAA ATAGCCTAAT AGCTCAACTA ACAGTAGATG TTTCATAACA GTTGTCAC --- 1325

                                        *
Domain a         135 --- CACGTG TCATGGTCCT GCTGG 1,325 [ES 15,363]
Domain c      1,312 [ES 15,350] CATGGTCCT GCTGGAGTT --- 1,409
```

<u>CAG clone 13 line 2</u>

```
(Alu/Alu junction)
                           •                                 *
Domain c         1 --- CACG TTTTTTTTTT TTTTTTTTTT TTTTTTTTTG AGACGGAGTC TCGCTCTGTC GAGTGCAGTG
Domain b 5'   146 [ES 8,984] TTTTTTTTTT TTTTTTTTTT TTTTTTTTTG AGACGGAGTC TCGCTCTGTC GAGTGCAGTG
                *                                 *
GCGGGATCTC GGCTCACTGC AAGCTCCGCC TCCCGGGTTC ACGCCATTCT CCTGCCTCAG CCTCCCAAGT AGCTGGGACT
GCGGGATCTC GGCTCACTGC AAGCTCCGCC TCCCGGGTTC ACGCCATTCT CCTGCCTCAG CCTCCCAAGT AGCTGGGACT
       *
CCGTTTTAGC ACAGGCGCCC GCCACTACGC CCGGCTAATT TTTTGTATTT TTAGTAGAGA CGGGGTTTCA CGGGATGGTC
CCGTTTTAGC ACAGGCGCCC GCCACTACGC CCGGCTAATT TTTTGTATTT TTAGTAGAGA CGGGGTTTCA CGGGATGGTC

TCGATCTCCT GACCTCGTGA TCCGCCCGCC TCGGCCTCCC AAAGTGCTGG GATTACAGGC GTGAGCCACC GCGCCCGGCC
TCGATCTCCT GACCTCGTGA TCCGCCCGCC TCGGCCTCCC AAAGTGCTGG GATTACAGGC GTGAGCCACC GCGCCCGGCC
                           •                *
TAAATAATGT TTTTTAGATT TGCCAAATTT GTACTGAACT CTAGAATGAC TAGTTAATCT TTTTCTCCTA TTGCAACCTT
TAAATAATGT TTTTTAGATT TGCCAAATTT GTACTGAACT CTAGAATGAC TAGTTAATCT TTTTCTCCTA TTGCAACCTT

CTAAATAATC ATTCAGCAAA ATAGCCTAAT AGCTCAACTA ACAGTAGATG TTTCATAACA GTTGTCACCT ATTGGGCAAC
CTAAATAATC ATTCAGCAAA ATAGCCTAAT AGCTCAACTA ACAGTAGATG TTTCATAACA GTTGTCACCT ATTGGGCAAC

ACTAGACGGC AGGCAGCATC T 636 [ES 16,272]
ACTAGACGGC AGGCAGCATC TAGTTAC --- 2,855

Domain b 5'       146 --- CCTCCCC ATAAGCGC 2,855 [ES 13,743]
Domain b 3'     2,848 [ES 13,736] ATAAGCGCCCCTCC --- 4,505
```

<u>CAG clone 13, line 3</u>

```
(Alu/Alu junction)

Domain c               1 --- CACGTG TTTTTTTTTT TTTTTTTTTT TTTTTTTTTG AGACGGAGTC TCGCTCTGTC
Domain b 5'    135 [ES 8,978] CACGTG TTTTTTTTTT TTTTTTTTTT TTTTTTTTTG AGACGGAGTC TCGCTCTGTC
                       *                              *
GAGTGCAGTG GCGGGATCTC GGCTCACTGC AAGCTCCGCC TCCCGGGTTC ACGCCATTCT CCTGCCTCAG CCTCCCAAGT
GAGTGCAGTG GCGGGATCTC GGCTCACTGC AAGCTCCGCC TCCCGGGTTC ACGCCATTCT CCTGCCTCAG CCTCCCAAGT
               *

AGCTGGGACT CCGTTTTAGC ACAGGCGCCC GCCACTACGC CCGGCTAATT TTTTGTATTT TTAGTAGAGA
AGCTGGGACT CCGTTTTAGC ACAGGCGCCC GCCACTACGC CCGGCTAATT TTTTGTATTT TTAGTAGAGA

CGGGGTTTCA CGGGATGGTC TCGATCTCCT GACCTCGTGA TCCGCCCGCC TCGGCCTCCC AAAGTGCTGG
CGGGGTTTCA CGGGATGGTC TCGATCTCCT GACCTCGTGA TCCGCCCGCC TCGGCCTCCC AAAGTGCTGG
```

```
       *                                        *
GATTACAGGC GTGAGCCACC GCGCCCGGCC TAAATAATGT TTTTTAGATT TGCCAAATTT GTACTGAACT CTAGAATGAC
GATTACAGGC GTGAGCCACC GCGCCCGGCC TAAATAATGT TTTTTAGATT TGCCAAATTT GTACTGAACT CTAGAATGAC
**                         *                                   *
TAGTTAATCT TTTTCTCCTA TTGCAACCTT CTAAATAATC ATTCAGCAAA ATAGCCTAAT AGCTCAACTA ACAGTAGATG
TAGTTAATCT TTTTCTCCTA TTGCAACCTT CTAAATAATC ATTCAGCAAA ATAGCCTAAT AGCTCAACTA ACAGTAGATG


TTTCATAACA GTTGTCACCT ATTGGGCAAC ACTAGACGGC AGGCAGCATC T 636 [ES 11,488]
TTTCATAACA GTTGTCACCT ATTGGGCAAC ACTAGACGGC AGGCAGCATC TAGTTA --- 2,848
```

```
                            *   *    *
Domain b 5'        135 --- GAGTCCTCCCCA 2848 [ES 13,922]
Domain b 3'   2,841 [ES 7,767] CCTCCCCATA AAGCG --- 4,527
```

CAG clone 13, line 4

```
                              (no homology)
                                   |
Domain c        132 --- GACCAGGATGG 821 [ES 14,737]
Chr. 3         822 [nt 36,813,626] TCTTTAGTGGCATCTCTGC --- 1,812

                                *    *
Chr. 3              822 --- GAGTCCTCCCCA 1,812 [nt 36,814,612]
Domain b      2,836 [ES 13,729] CCTCCCCATA AAGCG --- 4,527
```

CAG clone 13, line 5

(Alu/Alu junction)

```
                             *
Domain c        1 ---  ACACG TGTTTTTTTT TTTTTTTTTT TTTTTTTTTT TTGAGACGGA GTCTCGCTCT GTCGCCCAGG
Chr. 15  136 [nt 74,578,157] TGTTTTTTTT TTTTTTTTTT TTTTTTTTTT TTGAGACGGA GTCTCGCTCT GTCGCCCAGG
                 ***          *         *    *  *          *  *
 CTGGAGTGTA GTGGCACCAT CTCGGCTCAC TGCAACCTCC ACCTCCTGGG TTCAAGCTAT TCTCCTGCCT CAGCCTCCCA
 CTGGAGTGTA GTGGCACCAT CTCGGCTCAC TGCAACCTCC ACCTCCTGGG TTCAAGCTAT TCTCCTGCCT CAGCCTCCCA
                     * ****   * *     *  *            *       *       *               *
 AGTAGCTGGG ATTACAGGCA TGTACCATTG CACCTGGATA ATTTTTTGTA TATTTTAGTA GAGACAGGAT TTCACCATGT
 AGTAGCTGGG ATTACAGGCA TGTACCATTG CACCTGGATA ATTTTTTGTA TATTTTAGTA GAGACAGGAT TTCACCATGT
  *  **            *               *               *         *       *    *         *
 TGGCCAGGCT GGTTTTGAGC TCCCAACCTC AGGTAATCCG CCCGCGTCGG CCTCCTAAAG TGTTAGGATT ACAGGCATGA
 TGGCCAGGCT GGTTTTGAGC TCCCAACCTC AGGTAATCCG CCCGCGTCGG CCTCCTAAAG TGTTAGGATT ACAGGCATGA
        *
 GCCACCGAGC CCGGCTGTCT TTGTT 450 [ES 16,023]
 GCCACCGAGC CCGGCTGTCT TTGTT --- 1,953 [nt 74,579,973]


Chr. 15        136 --- AGGCCAAGGCAGGTGGATCAC 1,954 [nt 74,578,483]
Domain b                1,953 [ES 15,387] ACTCTC GGCATG --- 1,969
```

G4 clone 1, line 1

```
Domain c  1 --- TATAGCTAGC AAGCGGCCG 140
Domain a              133      GCGGCCGCTCTAGAACTAG  --- 1345

                                           *
Domain a          133 --- GATCGAGG GCGAGGGCGA GG 1345
Domain b                     1334  GCGAGGGCGA GGAGCTGTTCACC --- 2034
```

G4 clone 1, line 2

```
(Alu/Alu junction)
                   ********
Domain c    1 ---  TTTTTTTT TTTTTTTTTT TTTTTTTTTT TGAGACGGAG TCTCGCTCTG TCGCCCAGGC TGGAGTGCAG
Domain a               149  TTTTTTTTTT TTTTTTTTTT TGAGACGGAG TCTCGCTCTG TCGCCCAGGC TGGAGTGCAG

TGGCGGGATC TCGGCTCACT GCAAGCTCCG CCTCCCGGGT TCACGCCATT CTCCTGCCTC AGCCTCCCAA GTAGCTGGGA CTACAGGCGC
TGGCGGGATC TCGGCTCACT GCAAGCTCCG CCTCCCGGGT TCACGCCATT CTCCTGCCTC AGCCTCCCAA GTAGCTGGGA CTACAGGCGC

CCGCCACTAC GCCCGGCTAA TTTTTTGTAT TTTTAGTAGA GACGGGGTTT CACCGTTTTA GCCGGGATGG TCTCGATCTC CTGACCTCGT
CCGCCACTAC GCCCGGCTAA TTTTTTGTAT TTTTAGTAGA GACGGGGTTT CACCGTTTTA GCCGGGATGG TCTCGATCTC CTGACCTCGT

GATCCGCCCG CCTCGGCCTC CCAAAGTGCT GGGATTACAG GCGTGAGCCA CCGCGCCCGG CCT 451
GATCCGCCCG CCTCGGCCTC CCAAAGTGCT GGGATTACAG GCGTGAGCCA CCGCGCCCGG CCT --- 1294


Domain a  149 --- GGATCCACCG GTCGCCACCA TGGTGAGCAA GGGCGAGGAG GTCATCAAAG AGTTCATGCG
Domain b    1171  GGATCCACCG GTCGCCACCA TGGTGAGCAA GGGCGAGGAG GTCATCAAAG AGTTCATGCG

CTTCAAGGTG CGCATGGAGG GCTCCATGAA CGGCCACGAG TTCGAGATCG AGGGCGAGGG CGA 1294
CTTCAAGGTG CGCATGGAGG GCTCCATGAA CGGCCACGAG TTCGAGATCG AGGGCGAGGG CGATG --- 1896
```

G4 clone 1, line 3

```
(Alu/Alu junction)
                          *
Domain c     1 ---   A CGTGTTTTTT TTTTTTTTTT TTTTTTTTTT TTTGAGACGG AGTCTCGCTC TGTCGCCCAG GCTGGAGTGC
Domain b 5'     143  A CGTGTTTTTT TTTTTTTTTT TTTTTTTTTT TTTGAGACGG AGTCTCGCTC TGTCGCCCAG GCTGGAGTGC
                                                                                          *
AGTGGCGGGA TCTCGGCTCA CTGCAAGCTC CGCCTCCCGG GTTCACGCCA TTCTCCTGCC TCAGCCTCCC AAGTAGCTGG GACTACAGGG
AGTGGCGGGA TCTCGGCTCA CTGCAAGCTC CGCCTCCCGG GTTCACGCCA TTCTCCTGCC TCAGCCTCCC AAGTAGCTGG GACTACAGGG

CGCCCGCCAC TACGCCCGGC TAATTTTTTG TATTTTTAGT AGAGACGGGG TTTCACCGTT TTAGCCGGGA TGGTCTCGAT CTCCTGACCT
CGCCCGCCAC TACGCCCGGC TAATTTTTTG TATTTTTAGT AGAGACGGGG TTTCACCGTT TTAGCCGGGA TGGTCTCGAT CTCCTGACCT

CGTGATCCGC CCGCCTCGGC CTCCCAAAGT GCTGGGATTA CAGGCGTGAG CCACCGCGCC CGGCCTAAAT AATGTTTTTT AGATTGCCAA
CGTGATCCGC CCGCCTCGGC CTCCCAAAGT GCTGGGATTA CAGGCGTGAG CCACCGCGCC CGGCCTAAAT AATGTTTTTT AGATTGCCAA
                   *                                       *
ATTTGTACTG ACTCTAGAAT GACTAGTTAA TCTTTTTCTC CTATTGCAAC CTTCTAAATA TCATTCAGCA AAATAGCCTA ATAGCTCAAC
ATTTGTACTG ACTCTAGAAT GACTAGTTAA TCTTTTTCTC CTATTGCAAC CTTCTAAATA TCATTCAGCA AAATAGCCTA ATAGCTCAAC

TAACAGTAGA TGTTTCATAA CAGTTGTCAC CTATTGGGCA ACACTAGACG GCAGGCAGCA TCT 636
TAACAGTAGA TGTTTCATAA CAGTTGTCAC CTATTGGGCA ACACTAGACG GCAGGCAGCA TCT --- 2845


Domain b 5'     143 ---AGTCT CCTCCCCAT 2845
Domain b (center)      2838  CCTCCCCATA AGC --- 3038

                                         **   *
Domain b (center)    2838 --- TCG GGGAGGCGGGGAGGCGGGGGGAG 3038
Domain b 3'              3019     GGGAGGCGGGGAGGCGGGGGGAG ACCT --- 4187
```

G4 clone 1, line 4

```
(Alu/Alu junction)
                          **
Domain c     1 ---   A CGTGTTTTTT TTTTTTTTTT TTTTTTTTTT TTTGAGACGG AGTCTCGCTC TGTCGCCCAG GCTGGAGTGC
Domain b 5'     143  A CGTGTTTTTT TTTTTTTTTT TTTTTTTTTT TTTGAGACGG AGTCTCGCTC TGTCGCCCAG GCTGGAGTGC
                                                                                          *
AGTGGCGGGA TCTCGGCTCA CTGCAAGCTC CGCCTCCCGG GTTCACGCCA TTCTCCTGCC TCAGCCTCCC AAGTAGCTGG GACTACAGGG
AGTGGCGGGA TCTCGGCTCA CTGCAAGCTC CGCCTCCCGG GTTCACGCCA TTCTCCTGCC TCAGCCTCCC AAGTAGCTGG GACTACAGGG

CGCCCGCCAC TACGCCCGGC TAATTTTTTG TATTTTTAGT AGAGACGGGG TTTCACCGTT TTAGCCGGGA TGGTCTCGAT CTCCTGACCT
CGCCCGCCAC TACGCCCGGC TAATTTTTTG TATTTTTAGT AGAGACGGGG TTTCACCGTT TTAGCCGGGA TGGTCTCGAT CTCCTGACCT
```

```
CGTGATCCGC CCGCCTCGGC CTCCCAAAGT GCTGGGATTA CAGGCGTGAG CCACCGCGCC CGGCCTAAAT AATGTTTTTT AGATTGCCAA
CGTGATCCGC CCGCCTCGGC CTCCCAAAGT GCTGGGATTA CAGGCGTGAG CCACCGCGCC CGGCCTAAAT AATGTTTTTT AGATTGCCAA

ATTTGTACTG ACTCTAGAAT GACTAGTTAA TCTTTTTCTC CTATTGCAAC CTTCTAAATA TCATTCAGCA AAATAGCCTA ATAGCTCAAC
ATTTGTACTG ACTCTAGAAT GACTAGTTAA TCTTTTTCTC CTATTGCAAC CTTCTAAATA TCATTCAGCA AAATAGCCTA ATAGCTCAAC

TAACAGTAGA TGTTTCATAA CAGTTGTCAC CTATTGGGCA ACACTAGACG GCAGGCAGCA TCT 637
TAACAGTAGA TGTTTCATAA CAGTTGTCAC CTATTGGGCA ACACTAGACG GCAGGCAGCA TCT --- 2854


Domain b 5’          143 --- AGTCT CCTCCCCA 2854
Domain b (center)             2847 CCTCCCCATA AGC --- 3036

Domain b (center)              2838 --- GGGAGG CGGGGAGGCG G 3036
Domain b 3’                              3026  CGGGGAGGCG GGGGGAG ACCT --- 4192
```

G4 clone 1, line 5

```
Domain c          1 --- AGCTTGCCTT GAGTGCTTCA 29
Domain b             21 [ES 13,173] AGTGCTTCAGCCGCTACC --- 525
```

<u>G4 clone 6 line 1</u>

(FRT/FRT junction)

```
                                                         *
Domain c          8 --- TCCTATTC CGAAGTTCCT ATTCCGAAGT TCCTATTCTC TAGAAAGTAT AGGAACTTC 1347 [ES 14,664]
Domain a             1300 [ES 1] CGAAGTTCCT ATTCCGAAGT TCCTATTCTC TAGAAAGTAT AGGAACTTCA CC--- 3658

Domain a           1300 --- TCCCCCTT 3658 [ES 2,354]
Domain b         3656 [ES 9,497] CTTGACGG --- 4306
```

<u>G4 clone 6 line 2</u>

(Alu/Alu junction)

```
                                        ***
Domain c              1 --- TTTTTTTTTT TTTTTTTTTT TTTTTTGAGA CGGAGTCTCG CTCTGTCGCC CAGGCTGGAG
Chr. 8  [nt 127,768,533] 152 TTTTTTTTTT TTTTTTTTTT TTTTTTGAGA CGGAGTCTCG CTCTGTCGCC CAGGCTGGAG

TGCAGTGGCG GGATCTCGGC TCACTGCAAC CGCTGCCTCC CAGGTTCAAG TGATTCTCCT GCCTCAGCTT CCCGAGTAGC
TGCAGTGGCG GGATCTCGGC TCACTGCAAC CGCTGCCTCC CAGGTTCAAG TGATTCTCCT GCCTCAGCTT CCCGAGTAGC

                                             ***** *
TGGGATTATA GGCGTGCACC ACCATACCTG GCTAATTTTT TTTTTTTTT 346 [ES 14,244]
TGGGATTATA GGCGTGCACC ACCATACCTG GCTAATTTTT TTTTTTTTT TTTTT --- 744

Chr. 8             149 --- CTATCTTC 740 [nt 127,769,132]
Domain b          744 [ES 12,708] CCAAGAACA --- 1713
```

<u>G4 clone 6 line 3</u>

(Alu/Alu junction)

```
                                                   ******
Domain c                            1 --- TTTTTTTTTT TTTTTTTTTT TTTTTTGAGA CGGAGTCTCG CTCTGTCGCC CAGGCTGGAG
Chr. 8               161  [nt 127,768,550] TTTTTTTTTT TTTTTTTTTT TTTTTTGAGA CGGAGTCTCG CTCTGTCGCC CAGGCTGGAG
                    *                   *                               **
TGCAGTGGCG GGATCTCGGC TCACTGCAAC CGCTGCCTCC CAGGTTCAAG TGATTCTCCT GCCTCAGCTT CCCGAGTAGC
TGCAGTGGCG GGATCTCGGC TCACTGCAAC CGCTGCCTCC CAGGTTCAAG TGATTCTCCT GCCTCAGCTT CCCGAGTAGC
                    *                 *                   *
TGGGATTATA GGCGTGCACC ACCATACCTG GCTAATTTTT TTTTTTTTT GGTA 334
TGGGATTATA GGCGTGCACC ACCATACCTG GCTAATTTTT TTTTTTTTT GGTA TTTTT --- 738

Chr. 8               161 --- CTATCTTC  738 [nt 127,769,132]
Domain b            738 [ES 12,704] CCAAGAACA --- 1703
```

<u>G4 clone 6 line 4</u>

```
Domain b             1 --- GCTCGACCAG GATGGGCAC 678 [ES 13,001]
Chr. 8               673 [nt 42,825,440] GGGCACGTAC CATCATGC --- 2046

                                                        *
Chr. 8               673 --- TTTTT TTTTTGAGAC GGAG 2046 [nt 42,826,812]
Domain c          2,032 [ES 14,097] TTTTTGAGAC GGAGCGAGAC --- 2221
```

<u>G4 clone 6, line 5</u>

(Alu/Alu junction)

```
                                         *           *         **                    *
Domain b          8  --- CACGTG TTTTTTTTTT TTTTTCTTTT TTTTGAGACG GAGTCTCGCT CTGTCGCCCA GGCTGGAGTG CAGTGGCGGG
Chr. 16     151 [nt 85,971,297] TTTTTTTTTT TTTTTCTTTT TTTTGAGACG GAGTCTCGCT CTGTCGCCCA GGCTGGAGTG CAGTGGCGGG

ATCTTGGCTC ACAGCAACCT CTGCCTCCCA GGTTCACCCT CCTGAGTAGC TGGGACTACA GGCTTCTGCC ACTACATCTG GCTAATTTTT
ATCTTGGCTC ACAGCAACCT CTGCCTCCCA GGTTCACCCT CCTGAGTAGC TGGGACTACA GGCTTCTGCC ACTACATCTG GCTAATTTTT
                        *****
GTATTTTTAG TAGAGATGGG TTTTTTGTCA TGTTGGCCAG GCTCGTCTCA AACTCTTGGC CTCAAGTGAT CCGCCCACCC TGGCCTCCCA
GTATTTTTAG TAGAGATGGG TTTTTTGTCA TGTTGGCCAG GCTCGTCTCA AACTCTTGGC CTCAAGTGAT CCGCCCACCC TGGCCTCCCA
                                  *   *                            * * ** *   * *     **
AAGTGTTGGG GTATTTTTAG TAGAGATGGG TTTTTTGTCA TGTTGGCCAG GCTCGTCTCA AACTCTTGGC CTCAAGTGAT CCGCCCACCC
AAGTGTTGGG GTATTTTTAG TAGAGATGGG TTTTTTGTCA TGTTGGCCAG GCTCGTCTCA AACTCTTGGC CTCAAGTGAT CCGCCCACCC
*         *
TGGCCTCCCA ATTACAGGCA TGAG 423
TGGCCTCCCA ATTACAGGCA TGAG TCATGGA --- 1,347

Chr. 16       151 --- TGG ACTCCTATAT 1,347 [nt 85,972,493]
Domain b         1345 {ES 13,652] ATCACTCTCG GCA --- 1,362
```

<u>H3 clone line 1</u>

(FRT/FRT junction)

```
Domain c            1 --- TAGCT AAGTTCCTAT TCCGAAGTTC CTATTCTCTA GAAAGTATAG GAACTTC 1337 [ES 16,050]
Domain a       1,298 [ES 1,446] AAGTTCCTAT TCCGAAGTTC CTATTCTCTA GAAAGTATAG GAACTTCACC --- 10,881

Domain a       1298 --- CTCGCT CTG 10,881 [ES 11,045]
Domain b    10,879 [ES 15,013] CTGAGCACCC --- 10,984
```

<u>H3 clone line 2</u>

(FRT/FRT junction)

```
Domain c               1 --- TAGCT AAGTTCCTAT TCCGAAGTTC CTATTCTCTA GAAAGTATAG GAACTTC 763 [ES 16,050]
Domain a          1,297 [ES 1,446] AAGTTCCTAT TCCGAAGTTC CTATTCTCTA GAAAGTATAG GAACTTCACC --- 4,104

Domain a              1,295 --- TTTATAAGGG 4104 [ES 4242]
Domain b              4,103 [ES 14,162] GGTCCC --- 5063
```

<u>H3 clone line 3</u>

```
Domain c                1 --- GGCCGGCCAC 713 [ES 16,071]
Chr. 8              713 [nt 127253516] CCTCCACCCC --- 1073

Chr. 8        713 --- GGGGAAAAAGCAAAA 1073 [nt 127253864]
Domain b 5'        1073 [ES 13,532] AGGACAAGGA --- 1,387

Domain b 5'             1,387 --- CTCGCGGCCGC 1,486 [ES 13,837]
Domain b 3'                   1486 [ES 13,915] CTAGGGATAACAGG --- 2,633
```

<u>H3 clone line 4</u>

```
Domain c                1 --- GTCAGTGTGGAA  101 [ES 15,387]
Domain b 5'           101 [ES 13,640] GGAAGGAGGAG --- 168

Domain b 5'           101 ---  CACAGACCCT 168 [ES 13,704]
Domain b 3'            168 [ES 13,915] CTTAGGGATAACAG --- 1,310
```

<u>H3 clone line 5</u>

```
Domain c        1 --- AAATTCG 994 [ES 16,061]
Domain b            993   CGCATC --- 1,335
```

Supplementary Figure 7F

ATTCT clone line 1

```
                                           *
Domain c            4  --- GCAAACCGGGCGC 546 [ES 13,198]
Domain d          541 [ES 18,628]GGGCGCGGGGC --- 1,644


Domain d            541 --- AGCAAAGGTCTA 1644 [ES 20,104]
Domain a          1,641 [ES 8,404] TCTAACTGGATCTCA --- 3,116

Domain a          1,641 ---  AGGGGGCGGAGGGAA 3,116 [ES 7,301]
Domain b        3,107 [ES 11,948]GCGGAGGGAAAGACGCTTTGCAGCAAA --- 3,501
```

ATTCT clone line 2

```
Domain c            4  --- GCAAACCGGGCGC 543 [ES 13,198]
Domain d          538 [ES 8,404] GGGCGCGGGGC --- 1,640


Domain d            538 --- AGCAAAGGTCTA 1640 [ES 7,301]
Domain a          1,638 [ES 18,628] TCTAACTGGATCTCA --- 3,109

Domain a          1,638 ---  AGGGGGCGGAGGGAA 3,109 [ES 20,104]
Domain b        3,100 [ES 11,948] GCGGAGGGAA AGACGCT --- 3,493
```

ATTCT clone line 3

```
Domain c                  4 --- TCCCAAACCC GGCACCC 521 [ES 13,117]
Domain a                513 [ES 8,329] CC GGCACCCT GTCCTAC --- 1,473
```

(Alu/Alu junction)

```
                                                  **
Domain a             513 --- AAACC CACGTGTT TTTTTTTTTT TTTTTTTTTT TTTGAGACGG AGTCTCGCTC TGTCGCCCAG GCTGGAGTGC
Domain b        1,162  [ES 10,992] CACGTGTT TTTTTTTTTT TTTTTTTTTT TTTGAGACGG AGTCTCGCTC TGTCGCCCAG GCTGGAGTGC

 AGTGGCGGGA TCTCGGCTCA CTGCAAGCTC CGCCTCCCG GGTTCACGCC ATTCTCCTGC CTCAGCCTCC CAAGTAGCTG GGACTACAGG CGCCCGCCAC
 AGTGGCGGGA TCTCGGCTCA CTGCAAGCTC CGCCTCCCG GGTTCACGCC ATTCTCCTGC CTCAGCCTCC CAAGTAGCTG GGACTACAGG CGCCCGCCAC
                                *                                                                      *
 TACGCCCGGC TAATTTTTTG TATTTTTAGT AGAGACGGG GTTTCACCGT TTTAGCCGGG ATGGTCTCGA TCTCCTGACC TCGTGATCCG CCCGCCTCGG
 TACGCCCGGC TAATTTTTTG TATTTTTAGT AGAGACGGG GTTTCACCGT TTTAGCCGGG ATGGTCTCGA TCTCCTGACC TCGTGATCCG CCCGCCTCGG

 CCTCCCAAAG TGCTGGGATT ACAGGCGTGA GCCACCGCG CCCGGCCT 1473 [ES 9,294]
 CCTCCCAAAG TGCTGGGATT ACAGGCGTGA GCCACCGCG CCCGGCCT GTTCTT --- 2,506
```

ATTCT clone line 4

```
Domain c 5’      4 --- GCTGAGT CTCCTCCCCA 1,371 [ES 14,031]
Domain c 3’    1,364 [ES 14,051] CCTCCCCATA AGCGC --- 1,672

Domain c 3’      1,364 --- AAATAGCTAA CGTTGGGC 1,672 [ES 14,359]
Domain b                  1,673 [ES 11,527] GCTTGTTTG --- 2,488
```

ATTCT clone line 5

```
                                              *      *   **
Domain c               1 AGAATAGAAT TTTTGAGATG AAGTCTCTCT  30 [ES 12,313]
Chr. 9           [nt  134091299] T TTTTGAGATG AAGTCTCACTCTTGT --- [nt 134091842]

Chr. 9                 [nt 134091299] --- CCTCCCGAAG [nt 134091842]
Domain b                        548 [ES 12,263] GAAGAATGTT --- 588
```

Supplementary Figure 7. Template switch nucleotide overlaps. Template switching overlap homologies of read lines 1-5 at sequence resolution for ES microsatellite clones (A) $(CAG)_{102}$ clone 10 cells, (B) $(CAG)_{102}$ clone 13 cells, (C) G4 clone 1 cells, (D) G4 clone 6 cells, (E) H3 cells, (F) $(ATTCT)_{47}$ cells. For each line the template switching domains are indicated. Numbers, not in brackets followed or preceded by dashes (---) indicate the 5' or 3' end of a domain; numbers not in brackets alongside a nucleotide indicate the exact nucleotide position in the read; numbers in brackets correspond to positions in the ES map or to nonallelic nucleotide positions (GRCh38).
