# Supplementary material for: Microsatellite break-induced replication generates highly mutagenized extrachromosomal circular DNAs
Source: NAR Cancer. 2024 Jun 8;6(2):zcae027. doi: 10.1093/narcan/zcae027 (PMC11161834; doi:10.1093/narcan/zcae027)
Supplement: zcae027_Supplemental_Files [file zcae027_supplemental_files.zip › Supplementary Figure 8A-G mutations.pdf]

Figure 8A, B

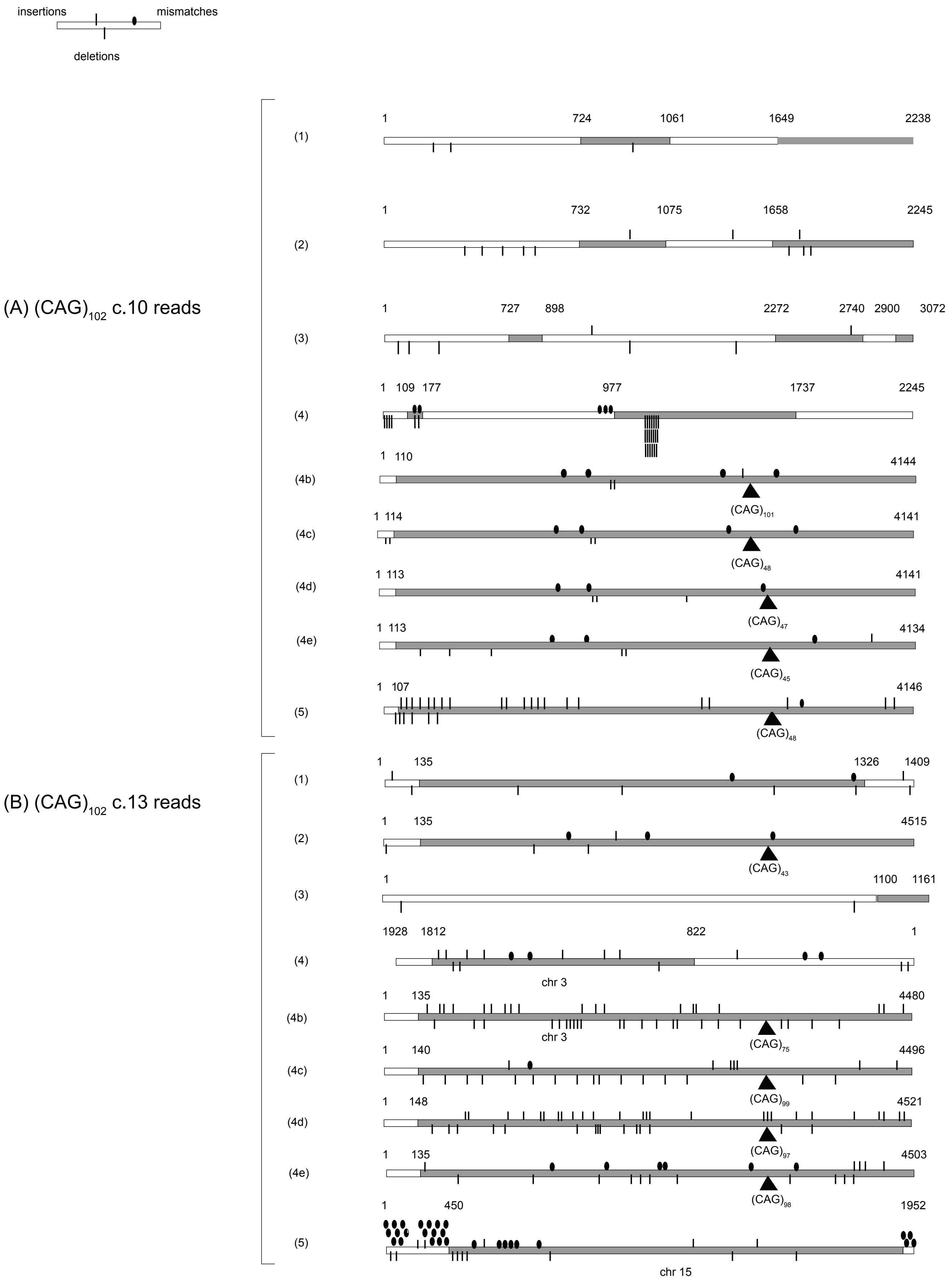

(Supplementary Figure 8 legend follows panel 8G)

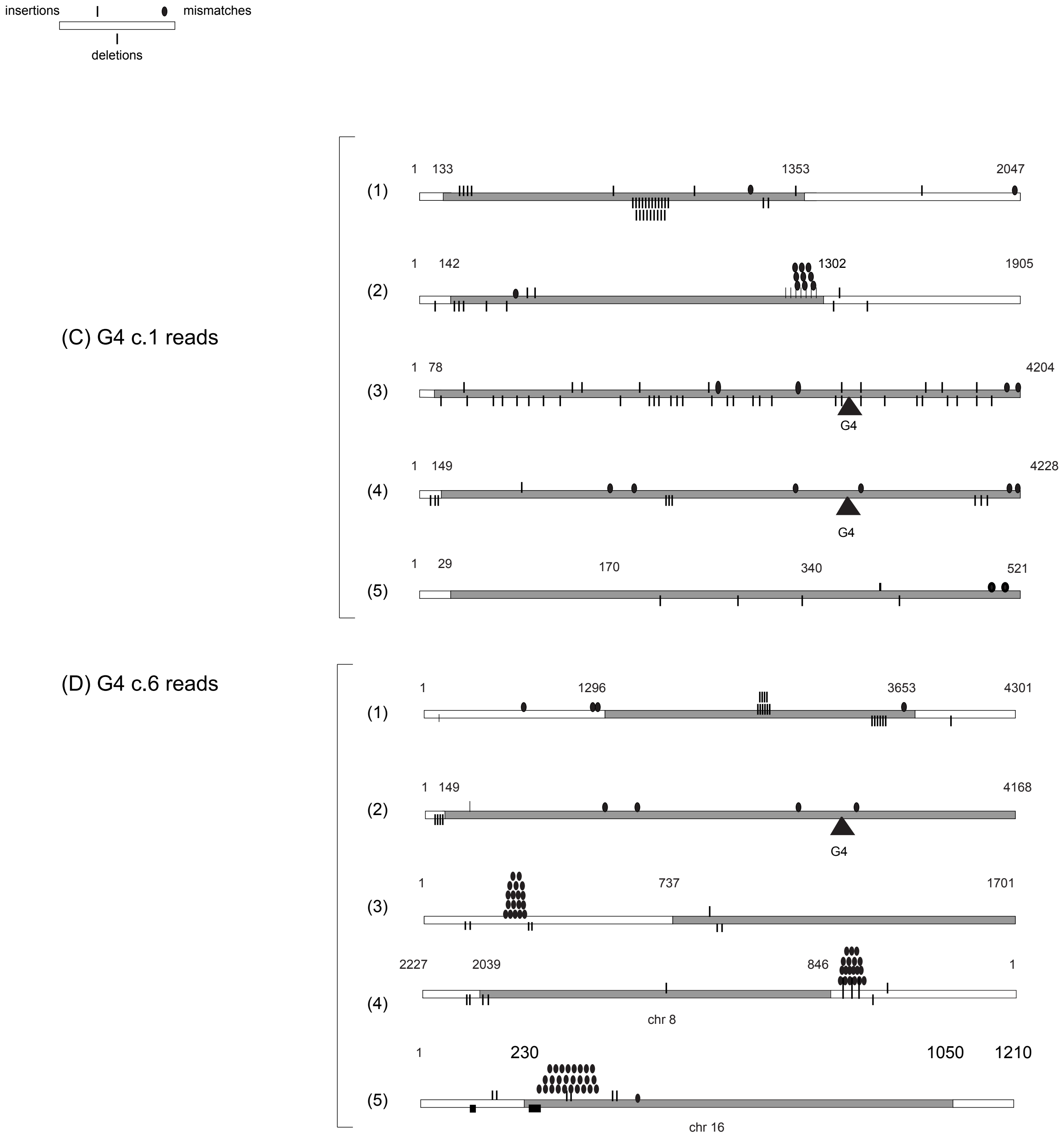

(Supplementary Figure 8 legend follows panel 8G)

Supplementary Figure 8E, F

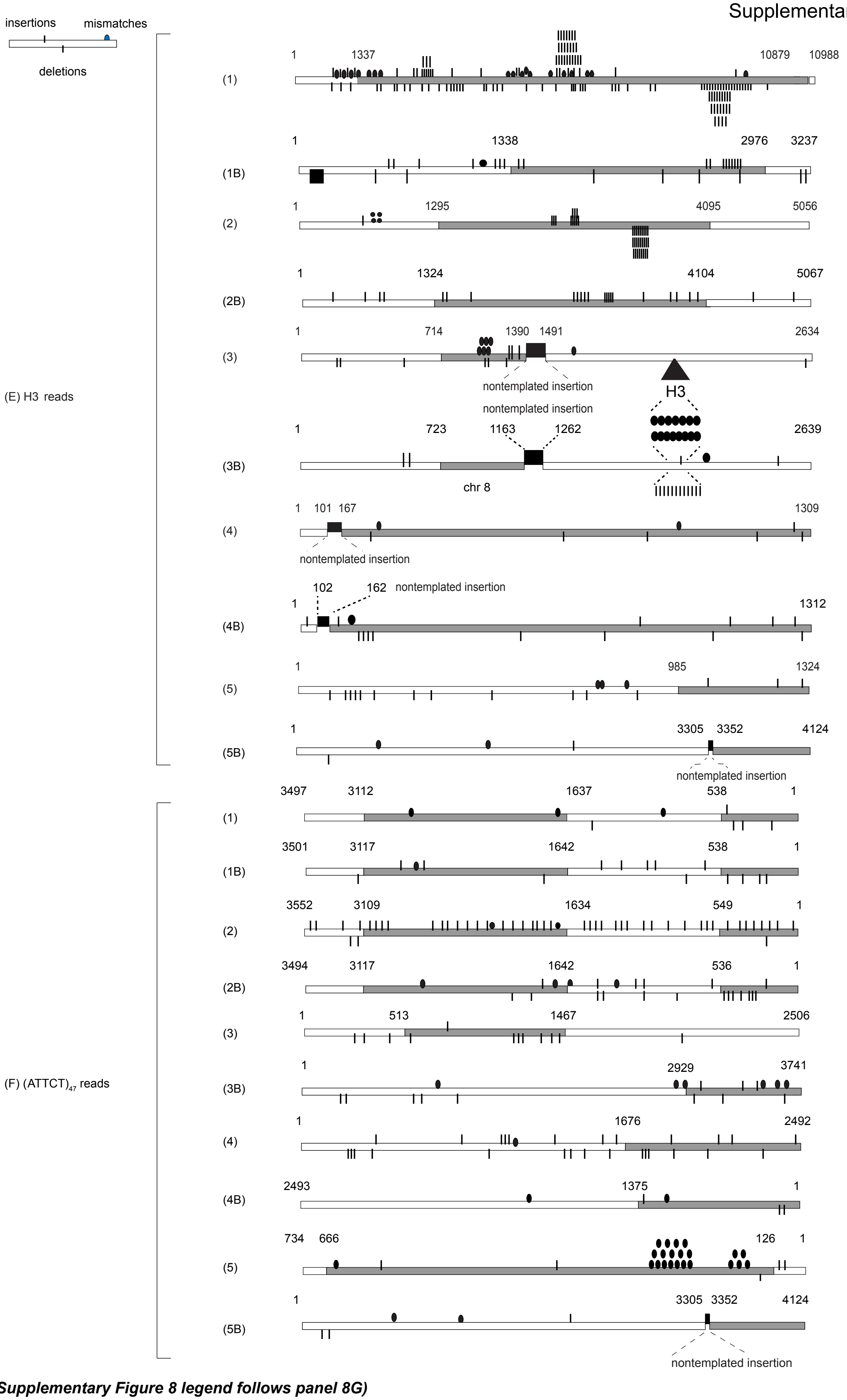

(Supplementary Figure 8 legend follows panel 8G)

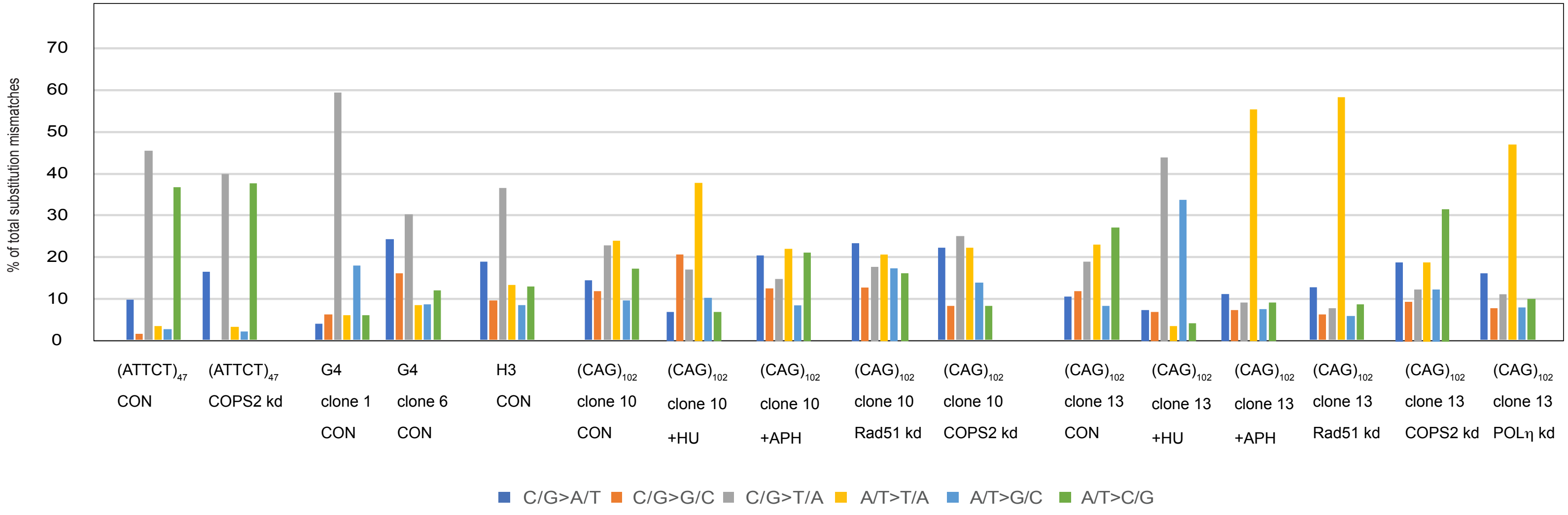

Supplementary Figure 8. Analysis of mutations. The approximate positions of mutations were mapped in BLAST for lines 1-5 of (A) $(CAG)_{102}$ clone 10 cells, (B) $(CAG)_{102}$ clone 13, (C) G4 clone 1, (D) G4 clone 6, (E) H3 lines, (F) $(ATTCT)_{47}$. Additional reads from the Ribbon overview viewpoint alignments (A) 4b-4e (neighboring line 4), (B) 4b-4e (neighboring line 4), (E) 1b, 2b, 3b, 4b, 5b (neighboring lines 1-5 respectively), (F) 1b, 2b, 3b, 4b, 5b (neighboring lines 1-5 respectively) illustrate the variability of mutagenesis patterns in similar regions of the ES. (G) The single base substitution signatures of the eccDNAs from $(CAG)_{102}$, G4, H3, and $(ATTCT)_{47}$ cell lines, as well as HU treated, and APH treated $(CAG)_{102}$ cells.
