# Supplementary material for: Microsatellite break-induced replication generates highly mutagenized extrachromosomal circular DNAs
Source: NAR Cancer. 2024 Jun 8;6(2):zcae027. doi: 10.1093/narcan/zcae027 (PMC11161834; doi:10.1093/narcan/zcae027)
Supplement: zcae027_Supplemental_Files [file zcae027_supplemental_files.zip › Supplementary Figure 9A-F CAG HU, APH.pdf]

(A)

$(CAG)_{102}$ clone 13
+ hydroxyurea

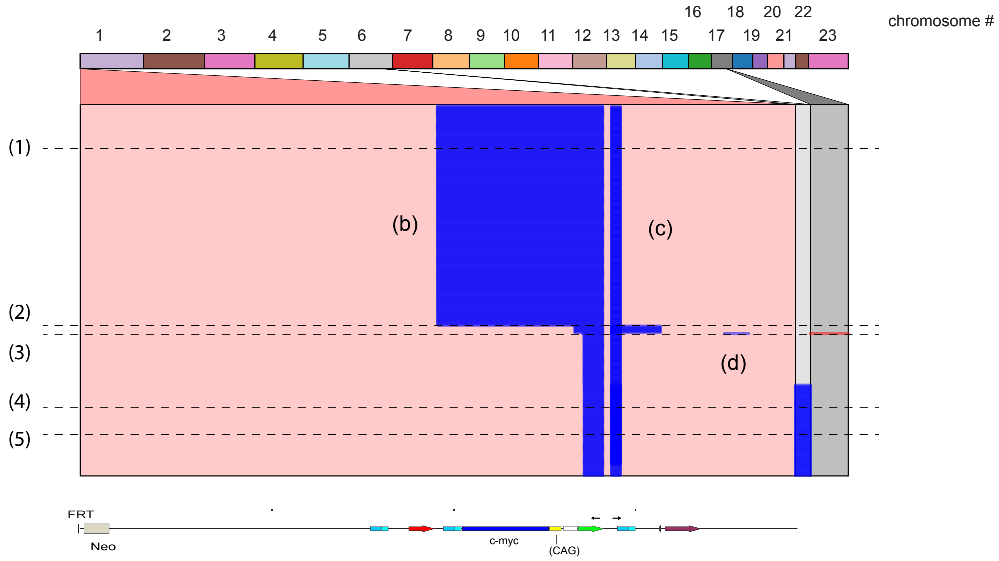

(B)

$(CAG)_{102}$ clone 13
+ aphidicolin

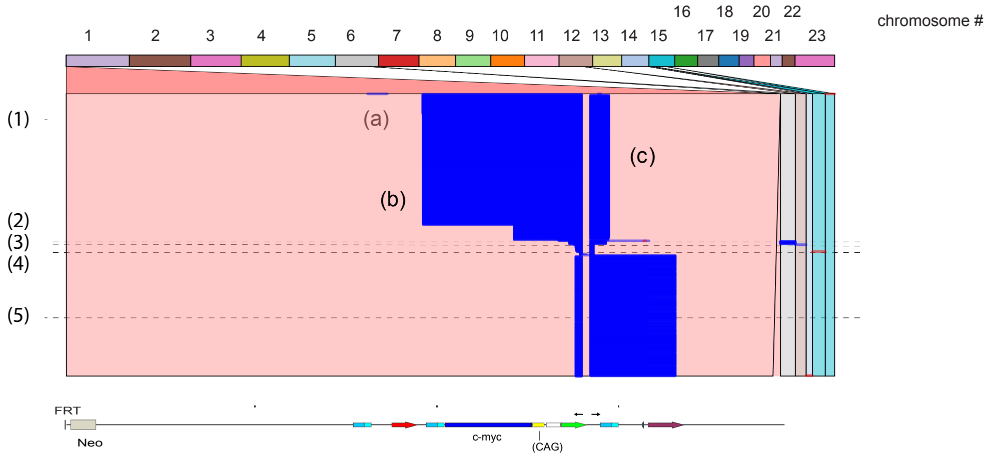

(Supplementary Figure 9 legend follows panel 9F)

Supplementary Figure 9C

$(CAG)_{102}$ clone 10 plus HU

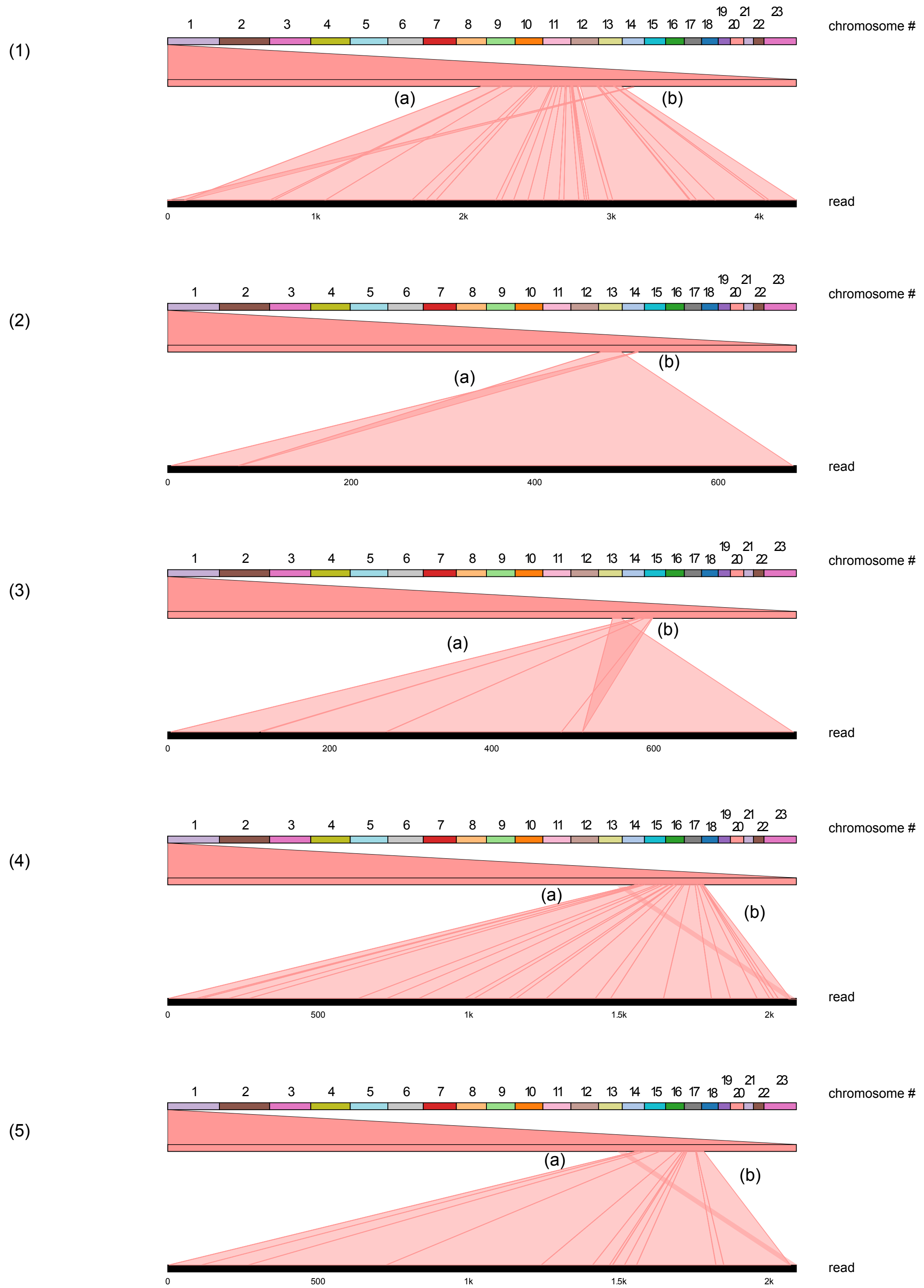

(Supplementary Figure 9 legend follows panel 9F)

Supplementary Figure 9D

$(CAG)_{102}$ clone 10 plus APH

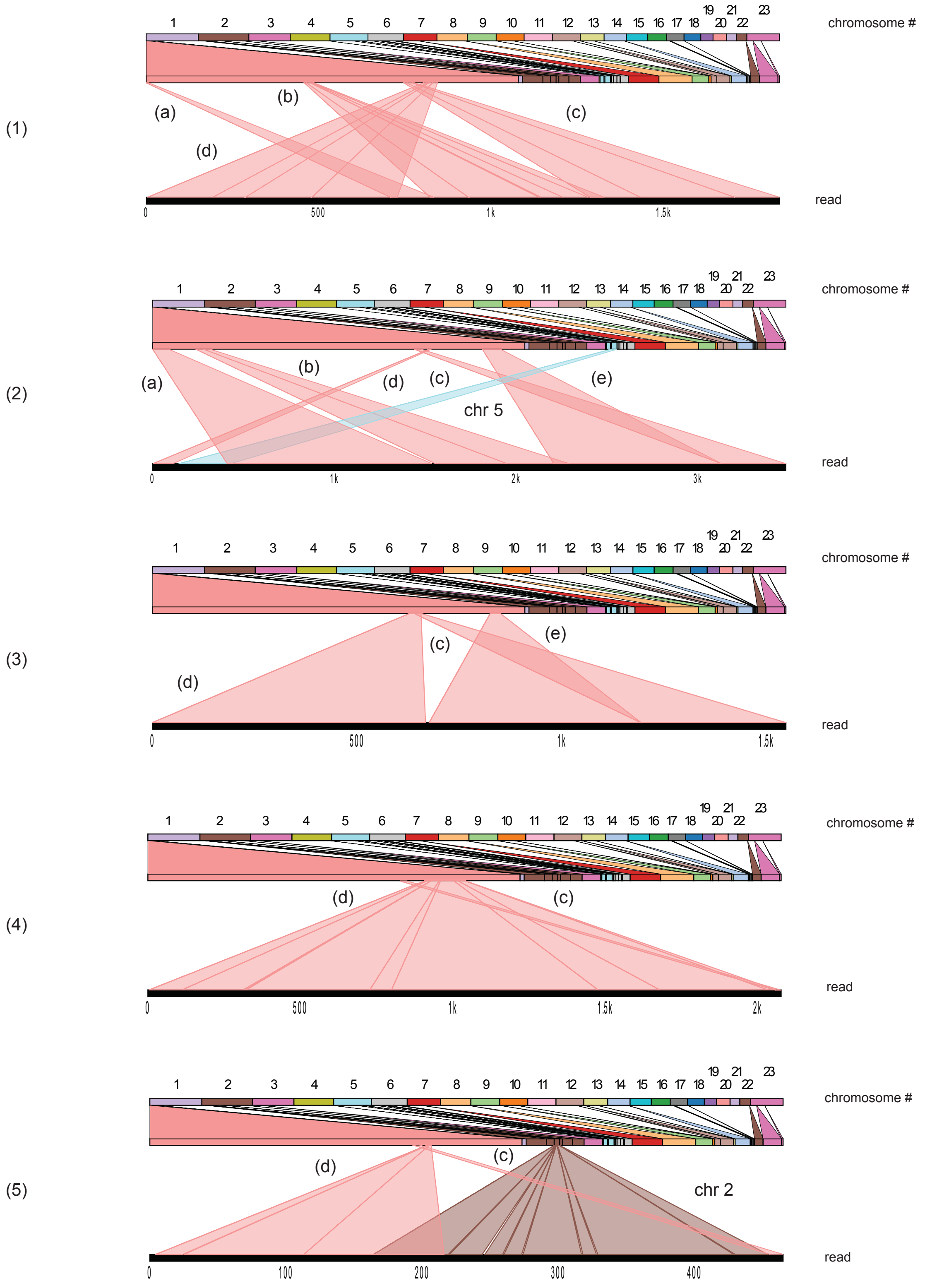

(Supplementary Figure 9 legend follows panel 9F)

Supplementary Figure 9E

(CAG)$_{102}$ clone 13 plus APH

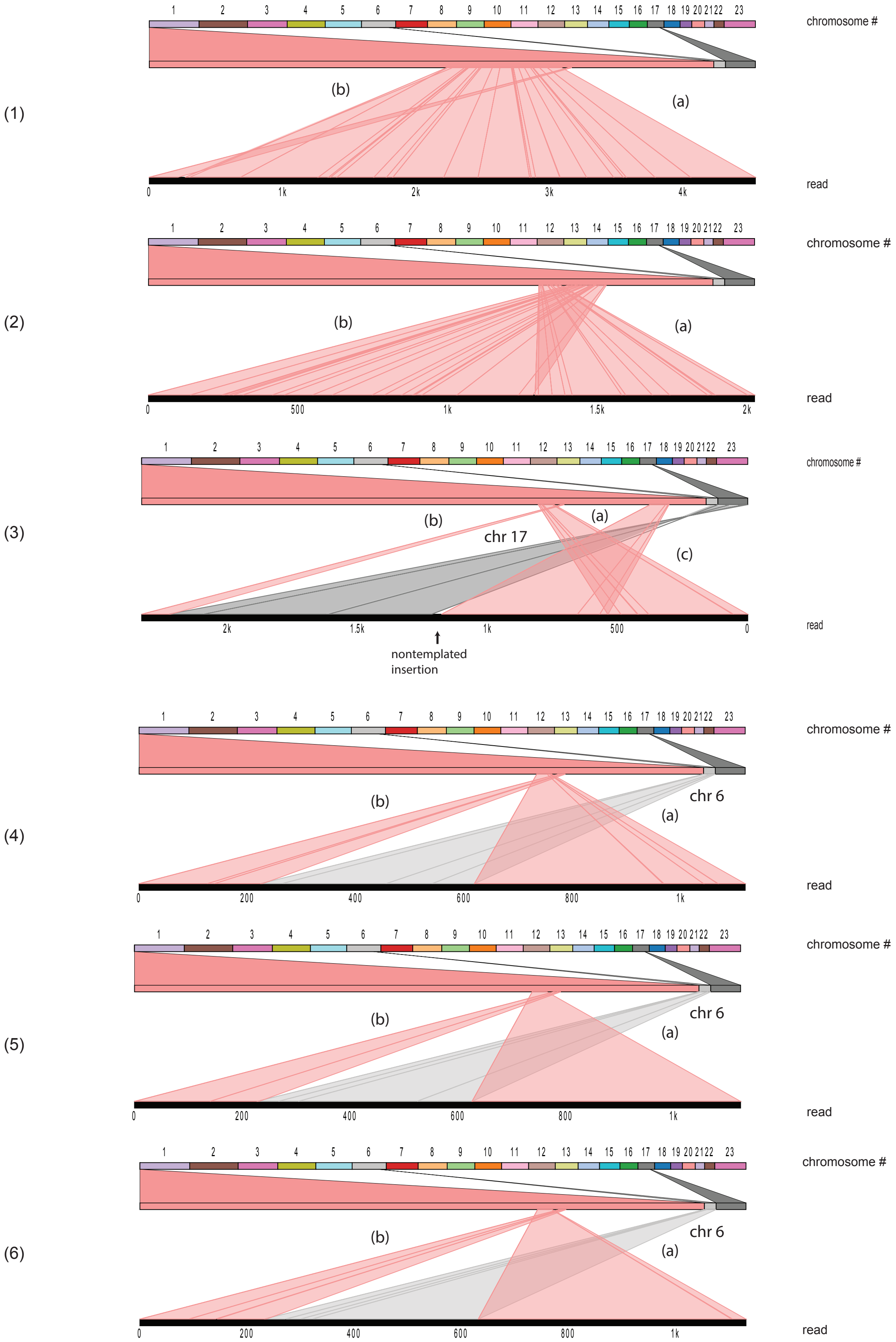

(Supplementary Figure 9 legend follows panel 9F)

(CAG)$_{102}$ clone 13 plus APH

Supplementary Figure 9F

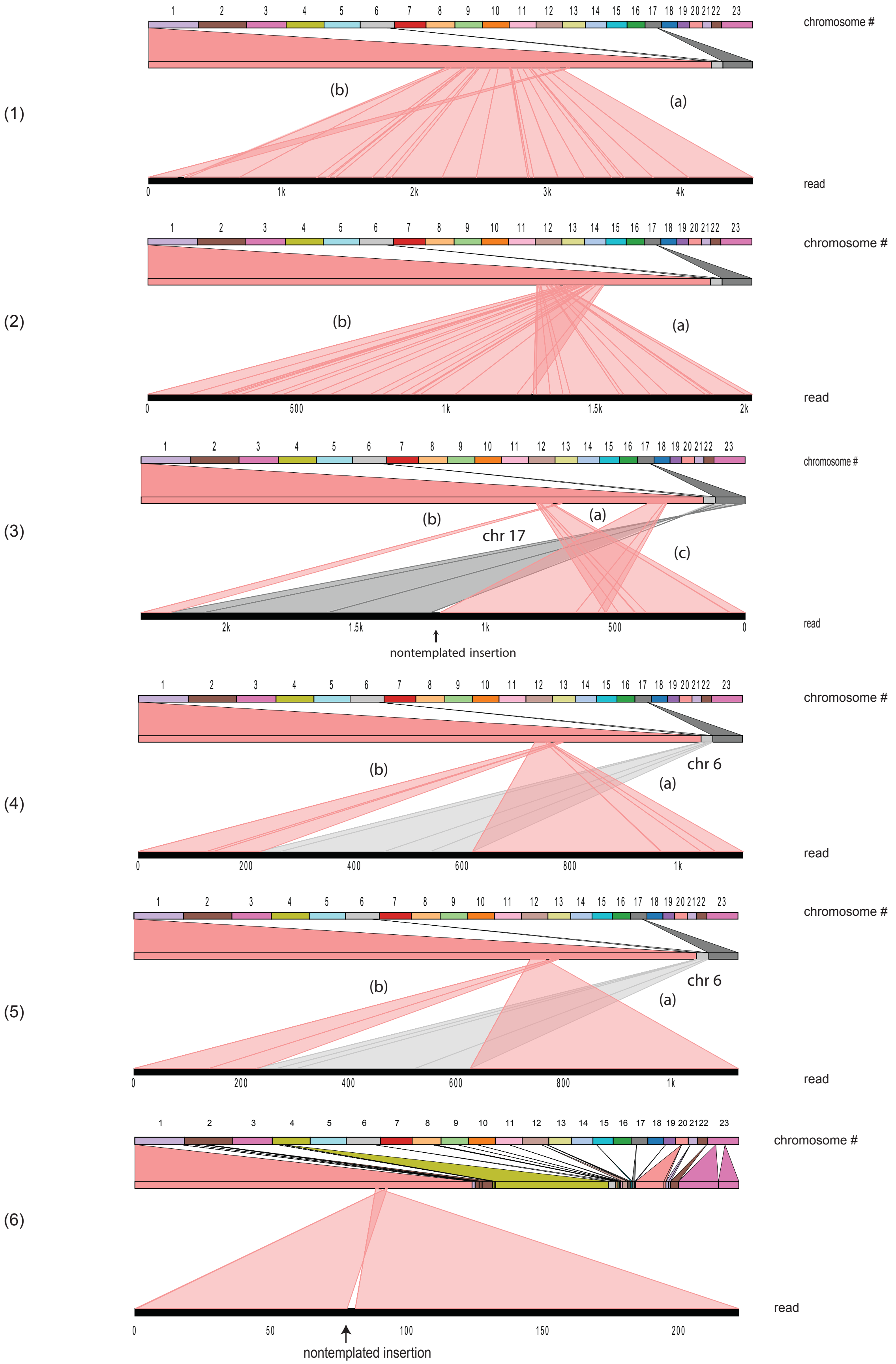

Supplementary Figure 9: Template switching patterns of inhibitor treated cells. Ribbon *Reference Viewport* alignment of iPCR reads from (CAG)$_{102}$ c.13 cells treated with (A) hydroxyurea (n=264 reads) or (B) aphidicolin (n=736 reads). Ribbon *Query viewport* view of reads from (C) (CAG)$_{102}$ clone10 cells treated with HU, (D) (CAG)$_{102}$ clone 10 cells treated with APH, (E) (CAG)$_{102}$ clone 13 cells treated with HU, (F) (CAG)$_{102}$ clone 13 cells treated with HU. Chromosome numbers are shown at the top; the lower heavy black line in each panel is the complete read. Heavier red lines indicate indels.
