# Supplementary material for: Microsatellite break-induced replication generates highly mutagenized extrachromosomal circular DNAs
Source: NAR Cancer. 2024 Jun 8;6(2):zcae027. doi: 10.1093/narcan/zcae027 (PMC11161834; doi:10.1093/narcan/zcae027)
Supplement: zcae027_Supplemental_Files [file zcae027_supplemental_files.zip › Supplementary Table 1.pdf]

Supplementary Table 1: si/shRNAs

siPOLη (SMARTpool)	AAACUGGCCUGAGGA
	CUAAGAAGUUAUGUCCAGAUCUU
	GCACUUACAUUGAAGGGUU
	GCAAUUAGCCCAGGAACUA
siCOPS2	CAAGACGAACCACUUGCUUAA
shRad51	CGCCCUUUACAGAACAGACUACUCGAGUAGUCUGUUCUGUAAAGGGCG
