# Supplementary material for: Microsatellite break-induced replication generates highly mutagenized extrachromosomal circular DNAs
Source: NAR Cancer. 2024 Jun 8;6(2):zcae027. doi: 10.1093/narcan/zcae027 (PMC11161834; doi:10.1093/narcan/zcae027)
Supplement: zcae027_Supplemental_Files [file zcae027_supplemental_files.zip › Supplementary Table 2.pdf]

Supplementary Table 2: iPCR primers

Sample name	Forward primer	Reverse primer
$(CAG)_{102}$ c.10	ATGTCCCGTCTGTTGTGTGACTCT	CGCTGCCGTCCTCGATGTTG
$(CAG)_{102}$ c.13	AAGCTTGCCTTGAGTGCTTC	AATTGTCCATGCCGAGAGTGATC
$(ATTCT)_{47}$	GGTATCTGTTTTCTATTTGTCTTCGGGAG	AGAATAGAATTTTTGAGATGAAGTCTCT
G4 c.1	AACACCTAAAGCTTGCCTTGAGTGCTTC	AACACCTAGTCCATGCCGAGAGTGATC
G4 c.6	ATATAGGAAAGCTTGCCTTGAGTGCTTC	ATATAGGAGTCCATGCCGAGAGTGATC
H3	ACGTAGCTAAGCTTGCCTTGAGTGCTTC	ACGTAGCTGTCCATGCCGAGAGTGATC
